# Identification of Molecules Responsible for Therapeutic Effects of Extracellular Vesicles Produced from iPSC-Derived MSCs on Sjo¨gren’s Syndrome

**DOI:** 10.14336/AD.2021.0621

**Published:** 2021-09-01

**Authors:** Hyemee Kim, Qingguo Zhao, Heather Barreda, Gagandeep Kaur, Bo Hai, Jong Min Choi, Sung Youn Jung, Fei Liu, Ryang Hwa Lee

**Affiliations:** ^1^Department of Molecular and Cellular Medicine, Institute for Regenerative Medicine, College of Medicine, Texas A&M University, College Station, Texas 77845, USA.; ^2^Department of Molecular and Cellular Biology, Baylor College of Medicine, Houston, TX 77030, USA.

**Keywords:** extracellular vesicles Mesenchymal stem/stromal cells, iPS cells, molecular profiling, Sjögren’s syndrome, sialadenitis

## Abstract

Recent research indicated that extracellular vesicles (EVs) derived from mesenchymal stem/stromal cells (MSCs) are a promising alternative to MSCs for immunomodulatory therapy. However, the contents of MSC-EVs would change as their parent MSCs change, hence the therapeutic efficacy of MSC-derived EVs (MSC-EVs) would largely depend on donors, tissue sources and culture conditions of MSCs. To overcome limitations of tissue-derived MSCs, we previously used MSCs derived from human induced pluripotent stem cells (iMSCs) to produce EVs and demonstrated their therapeutic potential in a mouse model of secondary Sjo¨gren’s Syndrome. Here, we further found that EVs from early-passage iMSCs had better immunomodulatory potency than EVs from late-passage iMSCs in TLR4-stimulated splenocytes and in a mouse model of primary Sjögren’s syndrome. Comparative molecular profiling using proteomics and microRNA sequencing revealed distinctive molecular profiles of iMSC-EVs with or without immunomodulation capacity. Amongst them, manipulation of TGF-β1, miR-21 and miR-125b levels in iMSC-EVs significantly affected their immunosuppressive effects. These findings would help improve our understanding of the molecular mechanism underlying iMSC-EV-mediated immunomodulation and further provide strategies to improve regulatory function of EVs for the treatment of immune-mediated diseases.

Lately, extracellular vesicles (EVs) derived from mesenchymal stem/stromal cells (MSCs) have been recognized as a promising alternative to MSCs for immunomodulatory therapy as MSC-derived EVs (MSC-EVs) recapitulate a broad range of the therapeutic effects shown by MSC treatment [1]. In our previous study, we also directly compared the therapeutic efficacy of bone marrow-derived MSCs (BM-MSCs) with their EVs in mouse models for type 1 diabetes and experimental autoimmune uveoretinitis. Our results showed that MSC-EVs were as effective as their parent MSCs in alleviating immune responses [2]. Consistent with our findings, several groups reported the immune-modulatory effects of MSC-EVs produced from various tissues, such as umbilical cord blood and adipose tissue [3-6], strongly supporting the therapeutic potential of MSC-EVs for the treatment of immune-mediated diseases.

However, since EVs carry proteins and genetic materials such as mRNAs and microRNAs (miRNAs) of their parent cells [7-10], the contents of MSC-EVs would change as their parent MSCs do. Hence, the therapeutic efficacy of MSC-EVs would largely depend on donors, tissue sources and culture conditions of MSCs. Indeed, our previous study showed that the contents of EVs change as their parent cells change due to different culture conditions [11]. In addition, we found that as biological properties of MSCs decline with *in vitro* expansion [12-14], and EVs from late-passage MSCs are less effective than those from early-passage MSCs [11]. These findings indicate that clinical application of tissue-derived MSCs and their EVs can be hindered by their limited expandability and considerable variations in biological properties caused by donors and culture conditions.

To overcome the limitations of tissue-derived MSCs, we had produced MSCs from transgene-free human induced pluripotent stem cells (iPSCs) with theoretically unlimited expandability using an optimized protocol that can be easily scaled up to produce a huge amount of standardized MSCs [15]. Recently, we directly compared the therapeutic efficacy of iPSC-MSCs (iMSCs) and their EVs with BM-MSCs in NOD mice with secondary Sjögren’s syndrome (sSS) and found that iMSC-derived EVs (iEVs) were as effective as their parent iMSCs in alleviating sSS [16]. Mechanistically, we found that iEVs suppressed the activation of immune cells and expression of pro-inflammation factors essential for SS progression *in vitro*, and infusion of iPSC-MSC EVs at the pre-disease stage decreased the lymphocyte infiltration in salivary glands (SGs) and serum levels of autoantibodies as MSCs did [16].

However, the molecular mechanism by which iEVs modulate the immune response is not fully understood. A previous study demonstrated that proteinase k treatment abolished the therapeutic effects of EVs [17]. Also, the knockdown of Alix, a component of the endosomal sorting complex required for transport, using siRNA in MSCs resulted in an 85% reduction of miRNAs in the secreted EVs and the loss of anti-scarring activity of these EVs [18]. In addition, we found that manipulation of EV contents directly affected their immunomodulatory effect *in vitro* and *in vivo* [11]. Therefore, the identification of proteins and RNAs responsible for the therapeutic effects of EVs is crucial to understanding the mechanism underlying EV-mediated immunomodulation. Nevertheless, as EVs carry numerous factors from their parent cells, defining therapeutic factors in MSC-EVs is quite challenging. In this regard, our previous study developed a strategy to identify key molecules responsible for EV-mediated immunomodulation through comparative molecular profiling between functionally different EVs produced from young and aged BM-MSCs [11]. With the same unbiased approach, we here identified distinctive molecular profiles of EVs from early- and late-passage iMSCs and further validated their roles in immunomodulation.

## MATERIALS AND METHODS

### iMSC Culture

The human iMSCs differentiated in our laboratory [15] were plated at a density of 500 cells per cm^2^ of growth area in complete culture medium [CCM; αMEM medium containing 17% (v/v) heat-inactivated fetal bovine serum (FBS, Atlanta Biologicals), Penicillin-streptomycin and L-glutamine] at 37 ºC and 5% CO_2_ and split at 70-80% confluence as previously optimized for BM-MSCs [19, 20]. To produce EVs from early-passage and late-passage iMSCs, passage 5 (P5) or P15 iMSCs were plated into 15-cm culture dishes at a density of 500 cells per cm^2^. When the cell density reached around 70-80% confluence, the cells were incubated with a serum-free and chemically defined medium optimized for Chinese hamster ovary cells (CD-CHO Medium, Invitrogen, Carlsbad, CA) [11]. After 6 h, the medium was replaced by fresh CD-CHO medium, and the conditioned medium was recovered at 48 hrs.

### Isolation of iMSC-EVs and characterization

For EV isolation, the conditioned medium was filtered to remove cellular debris (0.22 μm), and then EVs were isolated from the supernatant by ultracentrifugation (at 100,000 g for 16 h at 4°C) using Sorvall WX Floor Ultra Centrifuge with AH-629 36 ml swinging Bucket Rotor (Thermo Fisher Scientific, Waltham, MA). We pooled EVs from 8 to 10 cultures of MSCs (about 2.5 X 10^6^ cells per culture) into one sample and isolated EVs were resuspended with PBS at concentrations of 5 to 10 X10^10^/ml. The particle size and number of EVs were analyzed using the NanoSight LM 10 Nanoparticle Tracking Analysis System (Malvern, Malvern, UK). Also, the expression levels of EV surface markers CD9, CD63 and CD81 were analyzed by flow cytometry (CytoFLEX, Beckman coulter) using magnetic beads coated with anti-CD63 (human CD63 Isolation/Detection kit; Invitrogen), anti-CD63-FITC (clone H5C6; BD Biosciences), anti-CD81-PE (clone JS-81; Biosciences) and anti-CD9-FITC (clone eBioSN4; BD eBioscience). The isolated EVs were stored at -80°C.

### Protein profiling

Protein profiling of P5 and P15 iMSC-EVs was performed as described previously with modifications [21]. Briefly, EVs from 10 cultures of MSCs (about 2.5 X 10^6^ cells per culture) were pooled, boiled at 95 °C for 5 minutes in 50 mM ammonium bicarbonate and trypsinized for 16 hours. Peptide was extracted and subjected to a nanoLC-1200 (Thermo Scientific) coupled to Orbitrap Fusion mass spectrometer (Thermo Scientific) with ESI source. The peptides were loaded onto an in-house Reprosil-Pur Basic C18 (1.9 µm, Dr. Maisch GmbH, Germany) trap column (2 cm length, 100 µm i.d.) and separated by 5 cm column (150 µm i.d.) with a 75 min discontinuous gradient of 4-24 % of acetonitrile/0.1% formic acid at a flow rate of 800 nl/min. Precursor MS spectrum was scanned at 300-1400 m/z, 120k resolution at 400 m/z, 5x105 AGC target (50 ms maximum injection time) by Orbitrap. Top 3 second cycle time was applied to selected MS1 signal and filtered by Quadrupole (2 m/z isolation window, 15 s exclusion time), fragmented by HCD (32 normalized collision energy) and detected by Ion trap with rapid scan range (5x10^3^ AGC target, and 35 ms of maximum injection time). Obtained spectra were searched against the target-decoy Human RefSeq database (release 2020) in Proteome Discoverer 2.1 interface (PD 2.1, Thermo Fisher) with the Mascot algorithm (Mascot 2.4, Matrix Science). Dynamic modifications of the acetylation of N-terminus and oxidation of methionine were allowed. The precursor mass tolerance was confined within 20 ppm with fragment mass tolerance of 0.5 Da and a maximum of two missed cleavages was allowed. Assigned peptides were filtered with 1% false discovery rate (FDR) using percolator validation based on q-value. Label-free proteomics data were assigned to gene ID and calculated with the iBAQ algorithm for abundance by GPgrouper [22].

### Mouse splenocyte stimulation

Splenocytes were isolated from dissected spleen of adult (6 to 10 weeks old) male BALB/c mice (Jackson Laboratory, Bar Harbor, ME) euthanized with CO_2._ For T-cell activation, the splenocytes (2.5 × 10^5^ cells/well) were incubated with RMPI 1640 medium (Gibco) containing 5% FBS in the 96-well plates coated with anti-CD3 (Corning) [23] for 6 or 18 hours (hrs) with or without EVs. For LPS stimulation, splenocytes (5 × 10^5^ cells/well; 96 wells) were incubated with RMPI 1640 medium containing 5% FBS and 50 ng/ml LPS (Sigma, Saint Louis, MO) for 4 or 18 hrs with or without EVs. After stimulation, cell-free conditioned media were harvested to measure cytokine levels.

### Coculture of Human Mononuclear Cells with Salivary Gland Epithelial Cells

Human peripheral blood mononuclear cells (PBMCs) were purchased from Lonza. Healthy human salivary gland epithelial cells (SGECs) were isolated and cultured as we reported previously [24]. The SGEC-PBMC coculture experiment was based on a published protocol [25]. Briefly, SGECs were seeded at 1?×?10^5^ cells per well into 12-well plates and cultured in Keratinocyte serum-free medium (SFM, Life technology) with poly I:C (5?μg/ml, InvivoGen, tlrl-picw) for 12 hours to allow attachment, stimulation of autoantigen synthesis, and IL7 expression essential for SS progression. After removing the SFM and washing with PBS, 2?×?10^4^ PBMCs per well were added in LGM-3 lymphocyte growth medium (Lonza) containing 10% FBS and phytohemagglutinin-P (PHA-P, 5?μg/ml, Sigma-Aldrich, L8754) to activate T cells. After 4 days of coculture, SGECs and PBMCs were harvested together and analyzed for gene expression by qRT-PCR.

### ELISA

Mouse IFN-γ, IL-2, IL-6, IL-17 and TGF-β1 in the conditioned media of splenocytes, human TGF-β1 in transfected iMSC-EVs (1 x 10^10^ particle/ml), and Anti-La and Anti-Ro52 levels in serum from NOD.B10.H2^b^ mice were measured by commercial ELISA kits (R&D Systems, Minneapolis, MN; Signosis, Santa Clara, CA) according to the manufacturer’s protocol.

### Mouse model of primary Sjögren’s syndrome

Experiments were approved by the Texas A&M University Institutional Animal Care and Use Committee (IACUC). Four-month-old female NOD.B10.H2^b^ mice (Jackson Laboratory) were used as a model of primary Sjögren’s Syndrome (n=5/group). For treatment, PBS (100 μl) or iMSC-EVs (1.5 x 10^10^ particles in 100 μl PBS) derived from early- (P5) or late-passage (P15) iMSCs were injected into the tail vein twice a week for two weeks (Total four times). The dose of EVs was based on our previous study [11]. Two weeks after the last injection, submandibular glands (SMGs) and serum were collected. The areas of lymphocyte infiltrate were quantified from 3 H&E-stained sections from each of 5 SMGs.

### Real-time PCR analysis of mRNAs and miRNAs and miRNA profiling

RNA was extracted from SMGs of female NOD.B10.H2^b^ mice euthanized with CO_2_ two weeks after the last injection of EVs or PBS and transfected iMSCs and their EVs with Trizol (Invitrogen) and RNeasy Mini Kit (Qiagen, Hilden, Germany). The PCR probe and primer sets were purchased from Applied Biosystems (TaqMan Gene Expression Assay, Foster City, CA) and GAPDH was used as the reference RNA for mRNA. Total RNA was isolated from ultracentrifuged EVs (1x10^11^ particles) with the EZNA Total RNA Kit (Omega Bio-tek, Doraville, CA). miRNA sequencing with the total RNA was performed in LC Sciences (Houston, TX). P-value (<0.05) and fold change [log2 (fold change) ≥1] were used to determine the significant differential expression of miRNAs in RNA sequencing. For the confirmation of the miRNA sequencing data, miRNA expression levels of miR-21 and miR-125b were measured by using a TaqMan miRNA reverse transcription kit. The expression of miRNAs in iMSCs was normalized by the expression of U6B and the expression of miRNAs in EVs was normalized by miR-143 that was consistently expressed in all the conditions (early-passage EVs and late-passage EV) in our miRNA sequencing data. N = 3 for PCR analyses of cell cultures and 5 for mouse samples.

#### Cell transfection

Cells with 60% confluence were transfected with 20?nM siRNAs for control, TGF-β1 or TGF-β2, 20 nM miRNA inhibitors or mimics for control, miR-21-5p, or miR-125b-5p (Invitrogen) or 0.5 µg/ml DNA plasmids for control or TGF-β1 (OriGene, Rockville, MD) using RNiMax or Lipofectamine (Invitrogen) for 5?h. After transfection, cells were recovered with antibiotic-free CCM overnight for collecting EVs.

### Statistics

All data were analyzed using one way ANOVA followed by Dunnett’s or Tukey’s multiple-comparison tests. Statistical analysis and graphical generation of data were done with GraphPad Prism software (San Diego, CA).

**Figure 1.**
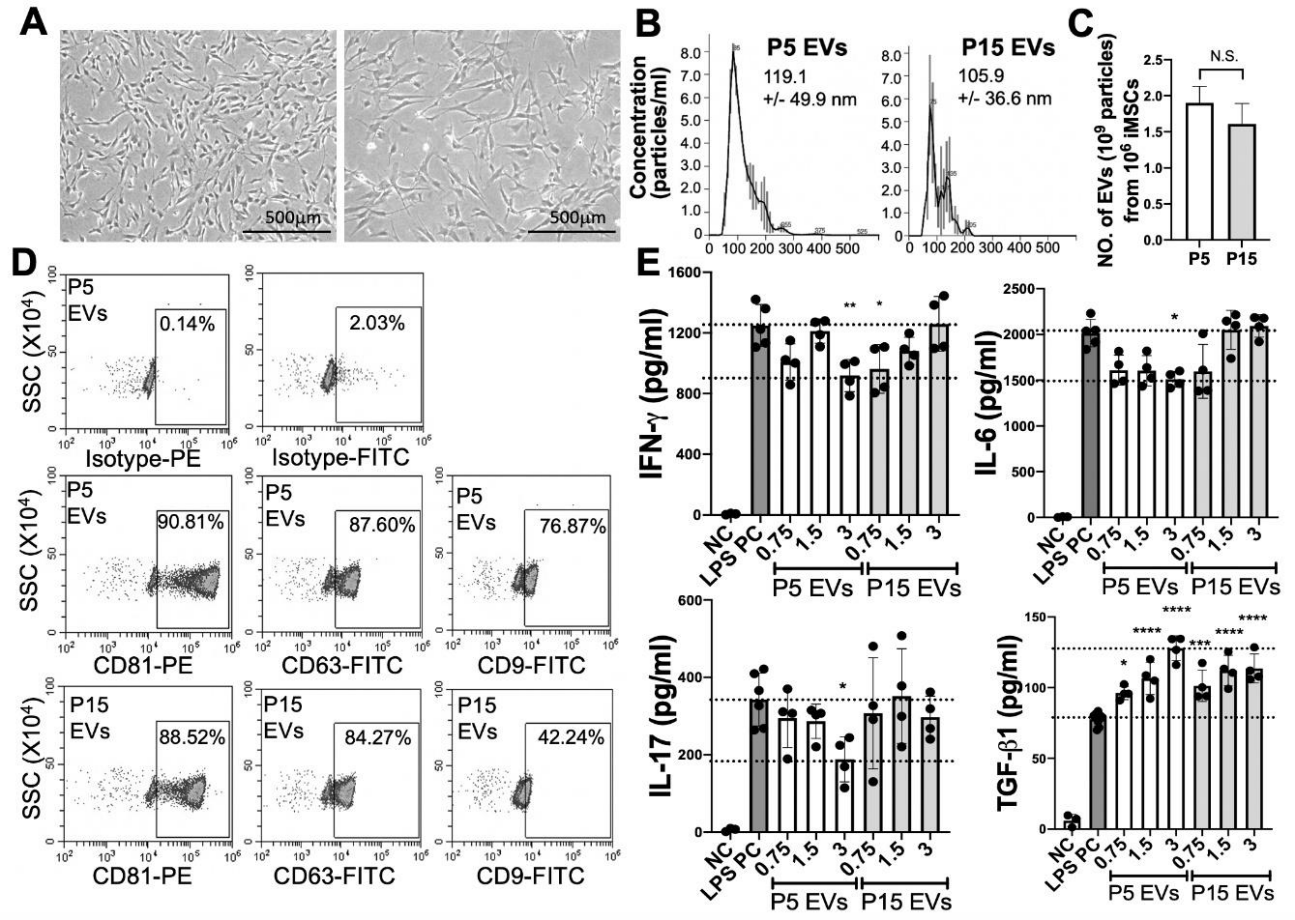
**Characteristics and *in vitro* immune modulation capacity of EVs from early- versus late-passage iMSCs**. (**A**) Representative morphology of early-passage (P5) iMSCs and late-passage (P15) iMSCs. (**B-C**) Particle sizes and production yields of P5 or P15 iMSC-EV were determined by nanoparticle tracking analysis. (**D**) Anslysis of EV surface markers CD9, CD63 and CD81 on P5 EVs and P15 EVs by flow cytometry. (**E**) Levels of IFN-γ, IL-6, IL-17, and TGF-β1 in conditioned medium of splenocytes stimulated with LPS (50 ng/ml) for 18 hrs with or without EVs (0.75 to 3 X10^9^ particles/ml) from P5 or P15 iMSCs were determined with ELISA. All data are presented as means ± SD (n=4-6). *: p < 0.05, **: p < 0.01, ***: p < 0.001, ****: p < 0.0001 by one-way ANOVA followed by Dunnett's test. NC: negative control; PC: positive control.

**Figure 2.**
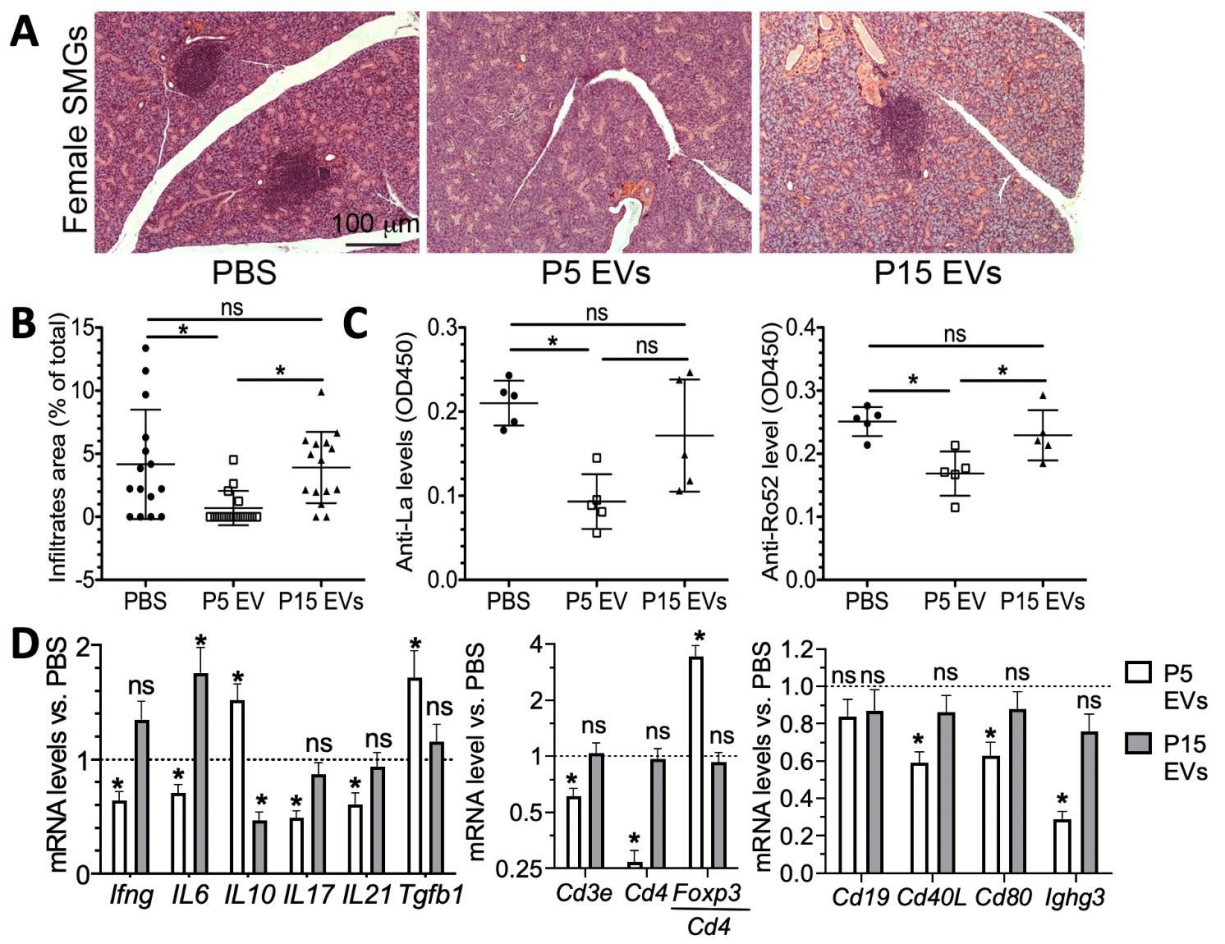
**Comparison of EVs derived from early- versus late-passage iMSCs in a mouse model of pSS**. EVs (1.5 × 10^10^ EVs/per mouse) derived from early- (P5) or late-passage (P15) iMSCs or vehicle control (PBS) were injected into the tail veins of NOD.B10-H2b mice twice a week for two weeks (n= 5). Two weeks after the last injection, SMG tissue and serum samples were collected. (**A**) Representative H&E staining of SMG tissue from NOD.B10-H2b females. (**B**) Quantification of the lymphocytic infiltration. The areas of lymphocyte infiltrate were quantified from three H&E stained sections from each of 5 SMGs. (**C**) Serum levels of anti-La and anti-Ro52 auto-antibodies were determined with ELISA. (**D**) Relative levels of mRNAs related to immune responses in submandibular glands were determined with qRT-PCR. All data are presented as means ± SD. ns: not significant, *: p < 0.05 by one-way ANOVA followed by Dunnett's or Tukey's test.

## RESULTS

### Comparison of immunomodulatory effects between early- and late-passage MSC-EVs

We compared EVs isolated from early-passage MSCs (passage 5 at seeding density 500 cells/cm^2^; population doubling 15; equivalent to Passage 2 at seeding density 100 cells/cm^2^) and late-passage MSCs (passage 15 at seeding density 500 cells/cm^2^; population doubling 40-45). P5 iMSCs show typical morphology of young MSCs (homogeneous, small, and spindle-shaped) while P15 iMSCs are heterogenous, large, flattened, and elongated (Fig. 1A). Between P5 and P15 EVs, the size, production yield, and levels of EV surface markers CD63 and CD81 are comparable (Fig. 1B-D), but the level of exosomal marker CD9 is lower in P15 iMSC-EVs (Fig. 1D). To evaluate the immunomodulatory effects *in vitro*, we used an *in vitro* culture system of LPS-stimulated splenocytes for the activation of Toll-like receptor 4 (TLR4) [26]. Our result showed that P5 iMSC EVs were more effective than P15 EVs in suppressing the secretion of Th1 and Th17 cytokines IFNγ, IL-6, and IL-17, as well as increasing the secretion of TGF-β1 (Fig. 1E).

The immunomodulatory effects of iMSC-EVs were further confirmed *in vivo* with a mouse model for primary Sjögren’s syndrome (pSS). pSS is a chronic autoimmune disorder characterized by immune cell infiltration and progressive injury to lacrimal and salivary glands, which precedes the major symptoms, keratoconjunctivitis sicca (dry eye) and xerostomia (dry mouth) in animal models [27, 28]. NOD.B10.H2b mice are reported to develop pre-clinical disease (sialadenitis) at 3 months old and clinical disease (hyposalivation) at 5-6 months old [29-31]. We also confirmed that in female NOD.B10.H2^b^ mice, the incidence of sialadenitis in SMGs is 40% at 3-month-old and 100% at 4-month-old (n = 5; data not shown). Therefore, P5 EVs or P15 EVs were infused into female 4-month-old NOD.B10.H2^b^ mice at the pre-disease stage via IV twice a week for two weeks. Two weeks after last injection, we collected SMGs and serum samples for the following analyses. H&E staining of SMG sections indicated that the size of lymphocyte infiltrates in SMGs in the P5 EV-treated group significantly decreased compared to PBS control group, whereas there was no significant difference between P15 EV and PBS groups (Fig. 2A-B). We examined the serum level of autoantibodies anti-Lo and anti-Ro52 by ELISA and found that only P5 EVs significantly decreased these two indexes (Fig. 2C). Since the pathogenesis of Sjögren’s syndrome involves activation of Th1 and Th17 cells preceded by activation of innate immune cells via TLRs [27, 28], we evaluated the effects of P5 EVs and P15 EVs on adaptive T cell immunity in SGs of NOD.B10.H2^b^ mice exhibiting pSS phenotypes. To compare effects of these two types of EVs on the composition and activation of lymphocytes infiltrated into SMGs, we examined the mRNA expression levels of various lymphocyte markers including Th1 marker IFN-γ, Th17 markers IL-17, IL-6 and IL-21, regulatory cytokines IL-10 and TGF-β1, pan-T cell marker Cd3e, helper T cell marker Cd4, Treg marker Foxp3, B cell marker Cd19, B/plasma cell marker Ighg3 and co-stimulating factors Cd40 and Cd80 by qRT-PCR. Consistent with our observations in LPS-stimulated splenocytes in Figure 1, P5 EVs significantly decreased the mRNA levels of markers for Th1 and Th17 cells and increased those of regulatory cytokines IL-10 and TGF-β1, whereas P15 EVs showed no significant effect (Fig. 2D). Also, P5 but not P15 EV treatment significantly decreased mRNA levels of B/plasma cell markers and co-stimulating factors and increased the relative mRNA expression of Treg marker Foxp3 normalized to Cd4 in SMGs compared to PBS group (Fig. 2D).

Together, the data demonstrate that EVs from early-passage iMSCs exhibit better immunosuppressive potency than those from late-passage iMSCs. Also, our data indicate that EVs can suppress the development of Th1 and Th17 cells likely by inhibiting the activation of antigen presenting cells (APCs) and T cells and by inducing the regulatory cytokine TGF-β1.

**Figure 3.**
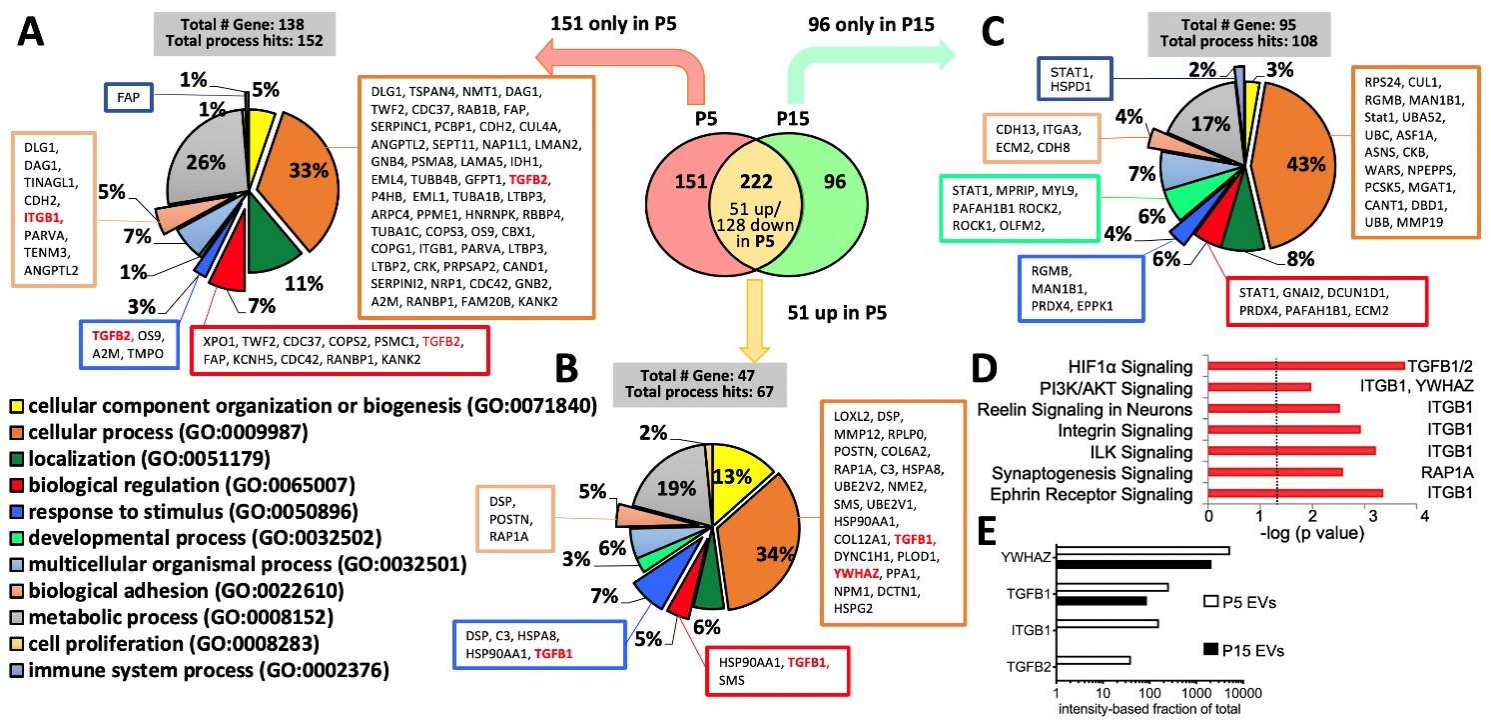
**Protein profiles of EVs from early- and late-passage iMSCs**. (**A**) Classification of proteins found only in P5 iMSC-EVs according to biological process. (**B**) Classification of proteins found in both but enriched (>1.5 folds) in P5 iMSC-EVs compared to P15 according to biological process. (**C**) Classification of proteins found only in P15 iMSC-EVs according to biological process. (**D**) Pathway analysis of all 202 proteins enriched in P5 EVs by IPA. (**E**) Intensities of proteins enriched in P5 EVs and their decrease in P15 EVs.

**Figure 4.**
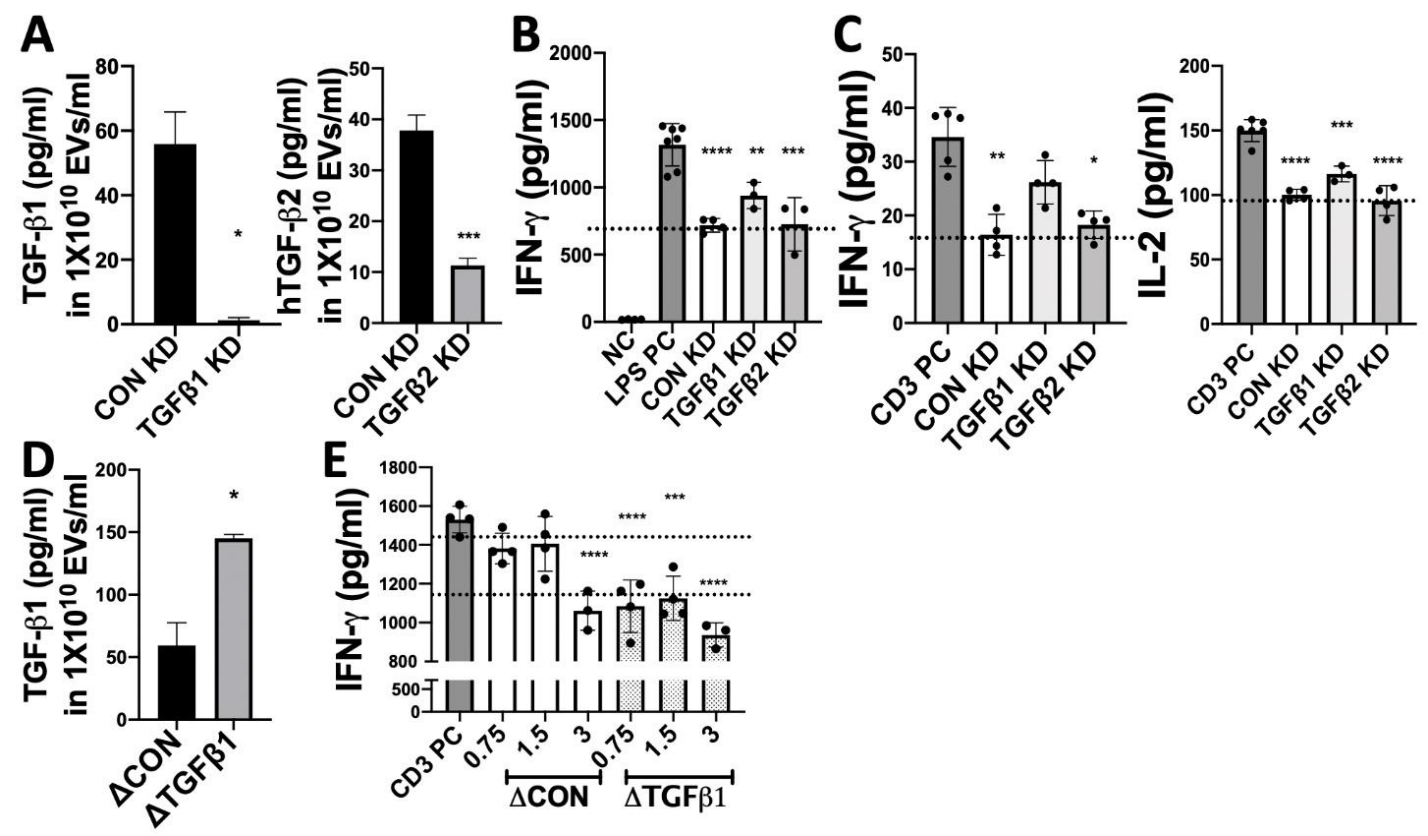
**Immunomodulatory effects of EVs from early-passage iMSCs with manipulated expression of TGF-β1 and TGF-β2**. (**A**) The level of TGF-β1 or TGF-β2 in EVs derived from P5 iMSCs transfected with siRNAs against TGF-β1 or TGF-β2 was determined with ELISA. (**B**) After treatment with EVs (3X10^9^ particles/ml) from control (CON) or TGF-β1/2 knockdown (KD) iMSCs, IFN-γ level in conditioned medium of LPS-stimulated splenocytes (18 hrs) was determined with ELISA. (**C**) Levels of IFN-γ and IL-2 in conditioned medium of splenocytes activated by anti-CD3 (6 hr) with or without EVs (3X10^9^ particles/ml) were determined with ELISA. (**D**) The level of TGF-β1 protein in EVs derived from P5 iMSCs transfected with control vector plasmids (ΔCON) or TGF-β1 plasmids (ΔTGFb1) was determined with ELISA. (**E**) After treatment (18 hrs) with EVs (0.75 to 3 X10^9^ particles/ml) from control or TGF-β1-overexpressing iMSCs at a series of concentrations, IFN-γ level in conditioned medium of splenocytes activated by anti-CD3 was determined with ELISA. All data are presented as means ± SD (n=4). *: p < 0.05, **: p < 0.01, ***: p < 0.001, ****: p < 0.0001 by one-way ANOVA with Dunnett’s test.

### Protein profiles of iMSC-EVs

In order to identify the distinctive protein profile of functionally-effective EVs in immunomodulation, we performed proteomics in EVs isolated from P5 iPSC-MSCs and P15 iPSC-MSCs. Proteomics identified about 470 proteins in MSC-EVs (Supplemental Table 1). Among them, 222 proteins were found in both P5 and P15 MSC-EVs and 151 proteins were found only in P5 MSC-EVs (Fig. 3A and B). As shown in Figures 1 and 2, P5 EVs were more effective in suppressing immune responses, suggesting that proteins enriched in P5 EVs could be related to the immunosuppressive functions. Therefore, we first analyzed proteins found only in P5 MSC-EVs (Fig. 3A) and upregulated >1.5 folds in P5 EVs (Fig. 3B) compared to P15 EVs using Protein Analysis Through Evolutionary and Relationship (PANTHER) software [32]. According to biological process analysis, the proteins enriched in P5 EVs are predominantly involved in the cellular process and metabolic process (Fig. 3A and B). Small percentages of the enriched proteins are involved in other biological processes including the response to cellular component organization or biogenesis, localization, biological regulation, response to stimulus, developmental process, and multicellular organismal process categories. PANTHER analysis of proteins found only in P15 EVs indicated that they are also predominantly involved in the cellular process and metabolic process (Fig. 3C). However, the percentage of proteins involved in developmental process increases. As indicated by the Ingenuity Pathway Analysis (IPA), proteins enriched in P5 EVs are related to the activation of inflammation regulatory pathways such as PI3K/Akt signaling (Fig. 3D). Among these proteins, TGF-β1, TGF-β2, YWHAZ and ITGB1 are abundant in P5 EVs (Fig. 3E). IPA analyses on proteins completely unique to P5 EVs or P15 EVs revealved huge numbers of pathways with P value < 0.05 (Supplemental Tables 2 and 3), which appears not as helpful as the IPA analyses of all proteins enriched in P5 EVs to narrow down the candidates.

**Figure 5.**
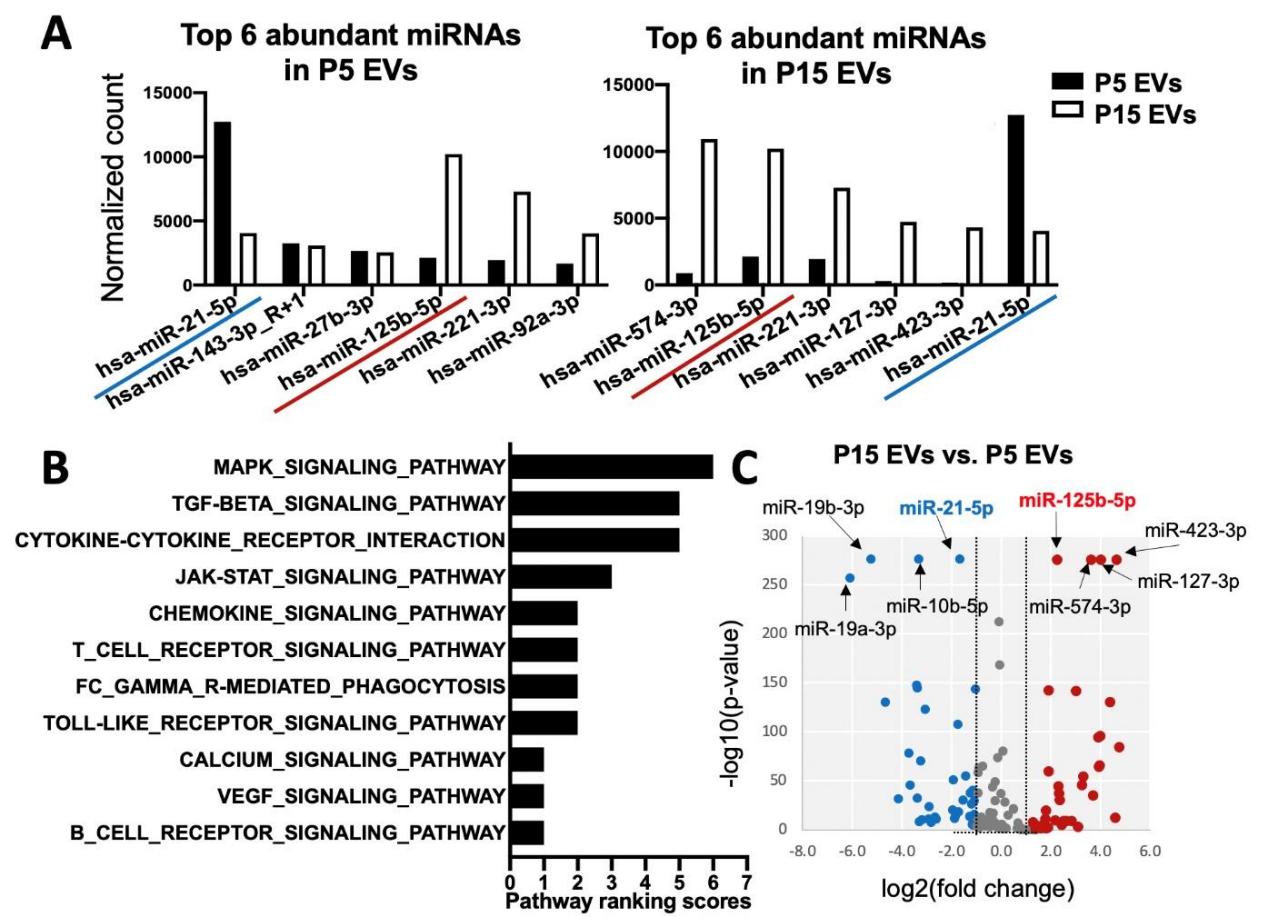
**Comparison of miRNA profiles of EVs from early- and late-passage iMSCs**. (**A**) Top 6 abundant miRNAs in P5 and P15 iMSC-EVs. (**B**) KEGG immune-mediated pathway ranking of predicted targets of the top 6 ranked miRNAs in EVs from early-passage iMSCs. (**C**) Volcano plot of the differentially expressed miRNAs in P15 EVs vs P5 EVs. The y-axis indicates the-log 10 of the P-values and the x-axis is the fold change (FC) (measured as the log2 transformed ratio of the expression between two groups).

Collectively, consistent with our observation that EVs from early- and late-passage iMSCs exhibit different immunomodulatory efficacy, proteomics data revealed that early- and late-passage iMSC-EVs have different protein profiles.

### Identification of proteins responsible for immune-modulatory effects of iMSC-EVs

To examine whether the proteins enriched in early-passage MSC-EVs are responsible for the EV-mediated therapeutic effect on pSS, we first selected TGF-β1 and TGF-β2 as candidates and validated their roles in immunomodulation. To manipulate levels of TGF-β1 and TGF-β2 in iMSC-EVs, we first transfected P5 iMSCs with corresponding siRNAs and successfully decreased these two protein levels in their EVs (Fig. 4A). The decrease of TGF-β1 but not TGF-β2 in iMSC-EVs partially abrogated the suppressive effects of EVs on the secretion of IFN-γ and IL-2 by LPS- or anti-CD3 stimulated splenocytes (Fig. 4B-C). In contrast, when we overexpressed TGF-β1 in EVs by transfection of cDNA plasmids in iMSCs (Fig. 4D), the EVs became more effective, and even lower doses of EVs were sufficient to suppress IFN-γ secretion in splenocytes activated by anti-CD3 (Fig. 4E). These results clearly demonstrate that TGF-β1 is one of major therapeutic factors responsible for the EVs-mediated immune suppression.

### miRNA profiles of iMSC-EVs

To identify the miRNA profile of functionally effective EVs in immunomodulation, we carried out miRNA sequencing with P5 EVs and P15 EVs (Supplemental Table 4). The top 6 ranked miRNAs found in P5 EVs (Fig. 5A) are involved in multiple pathways including MAPK, chemokine, TCR, Jak-STAT, calcium, and TLR signaling pathways (Fig. 5B and Supplemental Table 5) based on the KEGG pathway annotation of the predicted miRNA targets using the miRsystem [33]. Notably, miR-21-5p, the most abundant miRNA in P5 EVs, was significantly decreased in P15 EVs, while miR-125b-5p, the most abundant miRNA in P15 EVs, was conversely decreased in P5 EVs (Fig. 5C). Therefore, these two miRNAs were selected as the potential miRNA signature for further analysis based on expression levels in EVs (Fig. 5A), known target pathways (Fig. 5B) and *P* values (Fig. 5C).

**Figure 6.**
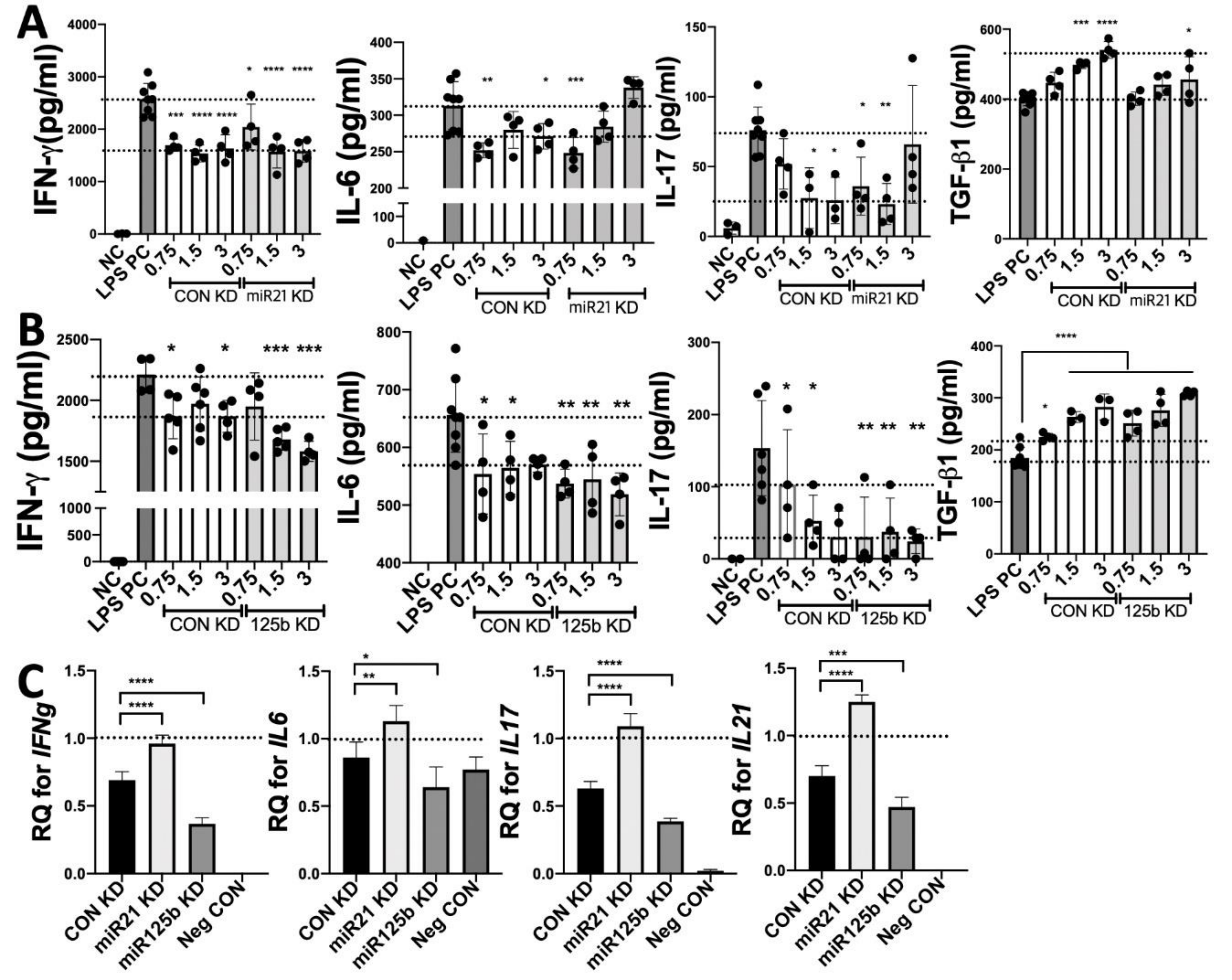
**Inhibition of miR-21 or miR-125b in early-passage iMSCs affects the immunomodulatory function of their EVs**. (**A-B**) After treatment with EVs (0.75 to 3 X10^9^ particles/ml) from P5 iMSCs transfected with control or miR-21/miR-125b inhibitors, levels of IFN-γ, IL-6, IL-17, and TGF-β1 in conditioned medium of LPS-stimulated splenocytes were determined with ELISA. (**C**) Relative mRNA levels of IFN-γ, IL-6, IL-17 and IL-21 in cocultured human SGECs and PBMCs treated with these EVs (3 X10^9^ particles/ml) were determined with qRT-PCR. All data are presented as means ± SD (n=4). *: p < 0.05, **: p < 0.01, ***: p < 0.001, ****: p < 0.0001 by one-way ANOVA with Dunnett’s test.

Silencing miR-21-5p in P5 iMSCs abrogated the inhibitory effects of their EVs on the secretion of IL-6, IFN-γ and IL-17 and the induction effects of their EVs on TGF-β1 production by LPS-stimulated splenocytes (Fig. 6A). In contrast, knockdown of miR-125b in P5 iMSCs enhanced these immunomodulatory effects of their EVs (Fig. 6B). The regulatory function of manipulated iEVs was further validated with a coculture system of human mononuclear cells with salivary gland epithelial cells (SGECs), which mimics interactions between SGECs and immune cells in SS patients [25, 34]. We confirmed the similar effects of manipulated EVs on the expression of IFN-γ, IL-6, IL-17 and IL-21 in cocultured human cells (Fig. 6C).

**Figure 7.**
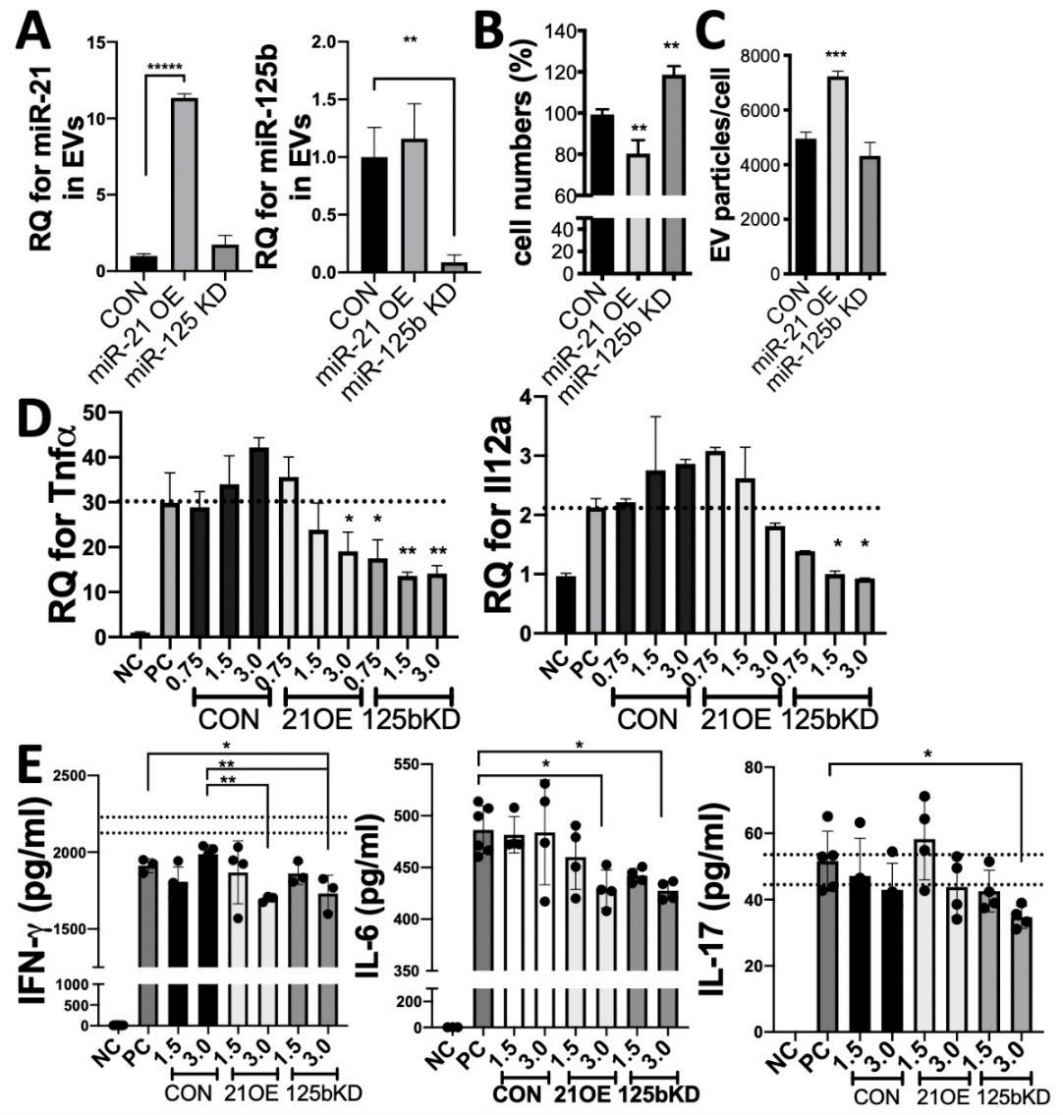
**Manipulation of miR-21 or miR-125b in late-passage iMSCs improves the immune regulatory function of their EVs**. (**A**) After transient transfection of miR-21 mimic or miR-125b inhibitor into P14 iMSCs, iEVs were isolated and levels of miR-21b and miR-125b in iEVs were determined with qRT-PCR. (**B-C**) The relative cell numbers and EV yields of iMSCs at 2 days after transfection with miR-21 mimic (OE) or miR-125b inhibitor (KD). (**D**) RT-PCR assays for TNFa and IL-12a in splenocytes stimulated with LPS for 4 hrs with or without EVs (0.75 to 3 X10^9^ particles/ml) from late-passage (P14) iMSCs transfected with target miRNA inhibitor or mimic. (**E**) IFN-γ, IL-6, and IL-17 ELISAs with conditioned medium of splenocytes stimulated with LPS for 18 hrs with EVs (0.75 to 3 X10^9^ particles/ml) derived from late-passage (P14) iMSCs transfected with target miRNA inhibitor or mimic. All data are presented as means ± SD (n=4). *p < 0.05, **p < 0.01, ***p < 0.001, ****p < 0.0001 by one-way ANOVA with Tukey’s multiple comparisons test.

To further validate roles of miR-21 and miR-125b in EV-mediated immune modulation, we transfected miR-21 mimic or miR-125b inhibitor into late-passage (P14) iMSCs to manipulate the level of miR-21 and the activity of miR-125b in their EVs and further examined regulatory function of their EVs with LPS-stimulated splenocytes. Our data showed that transient transfection of miRNA mimic or inhibitor in iMSCs significanly affected the levels of target miRNAs in EVs (Fig. 7A). However, in the case of miR-125b inhibitor, qRT-PCR may largely measure the inhibitory effect of the miRNA inhibitors in EVs as miRNA inhibitors are not expected to decrese the endogenous level of target miRNAs [35]. Interestingly, the effects of manipulation of miR-21 level and miR-125b activity were distinct on late-passage iMSCs and their particle release. We found that transient transfection of miR-21 mimic suppressed the proliferation of late-passage iMSCs while inducing an increase of EV particle release (Fig. 7B-C). In contrast, inhibition of miR-125b in iMSCs significantly increased the proliferation within 24 hrs and slightly inhibited EV production (Fig. 7B-C). Nevertheless, while control EVs from late-passage iMSCs were ineffective in suppressing Th1 and Th17 cytokine secretion in LPS-stimulated splenocytes, both manipulated EVs became effective in suppressing Th1 and Th17 cytokine secretion (Fig. 7D-E). Notably, the mRNA levels of TNFa were significantly suppressed by both manipulated EVs at 4h after LPS stimulation (Fig. 7D), indicating their direct inhibitory effect on TLR4 signaling.

Collectively, these data suggest that miR-21-5p is responsible for the EV-mediated inhibition on the expression of genes downstream of TLR4-signaling, whereas miR-125b interferes the immune regulatory function of EVs.

## DISCUSSION

Our data demonstrate that EVs derived from early-passage iMSCs more effectively suppress the expression of Th1 and Th17 cytokines in splenocyte cultures and increase the regulatory cytokine TGF-β1 compared to late-passage MSC-EVs. Similarly, EVs from early-passage iMSCs were more effective in suppressing the progression of pSS in a mouse model. Unbiased proteomic and RNA sequencing revealed distinct molecule profiles of these EVs: TGF-β1, TGF-β2, YWHAZ, ITGB1 and miR-21 are enriched in early-passage iMSC EVs, while miR-125b is enriched in late-passage iMSC EVs. Importantly, manipulation of TGF-β1, miR-21 and miR-125b in early-passage iMSCs significantly changed the suppressive effect of their EVs on Th1 and Th17 cytokine production. Furthermore, overexpression of miR-21 and inhibition of miR-125b in late-passage iMSCs improved the regulatory function of their EVs. Therefore, our strategy of comparative molecular profiling of iEVs herein reveals TGF-β1 and miR-21 as key effectors mediating the EV-mediated immunomodulation and miR-125b as a negative regulator.

In our previous study, the same strategy employing comparative molecular profiling identified TGF-β1, PTX3, let-7b-5p or miR-21 as key molecules mediating the therapeutic effects of BM-MSC-EVs in mice with ocular SS [11]. Similarly, TGF-β1 and miR-21 are enriched in early-passage iMSC-EVs. TGF-β1 is an anti-inflammatory cytokine capable of suppressing TLR and TCR signaling [36]. It can also induce miR-21 expression, which can target Smad7 and thereby enhance TGF-β-SMAD signaling [37]. Furthermore, miR-21 can directly inhibit Th1 polarization by limiting the activation of the IL-12/IFN-γ pathway [38]. Consistent with these findings, when we manipulated the expression level of TGF-β1 in iMSCs, the capacity of their EVs in suppressing TLR and TCR downstream genes was affected. Also, EVs from miR-21 knockdown young iMSCs failed to suppress IFN-γ secretion in LPS stimulated splenocytes and could not induce TGF-β1 expression as efficient as control EVs. Furthermore, miR-21 overexpression in late-passage iMSCs restored these immunomodulatory effects of EVs. Proteomics also revealed that early-passage iMSC-EVs contain high levels of YWHAZ and ITGB1. It has been shown that both YWHAZ and ITGB1 are highly expressed in immune suppressor cells, including Tregs and M2 macrophages, and are responsible for the resolution of chronic inflammatory arthritis [39]. Notably, exosomal YWHAZ derived from tumor cells can be transmitted to tumor-infiltrating T cells and impaired their antitumor activity by enhancing their differentiation to Tregs [40], while a secretory peptide derived from YWHAZ inhibits transendothelial migration of T cells in a mouse model of virally induced Sjögren’s syndrome [41]. In addition, it is possible that ITGB1 activates PI3K/AKT pathway after epithelial damage and inhibits epithelial cell inflammation through repressing ROS production and innate immunity [42]. Moreover, all these proteins may collaborate with miR-21 to activate PI3K/AKT signaling that inhibits JNK1 signaling and consequent apoptosis and inflammation [43].

Different to our previous findings in EVs from bone marrow MSCs, miR-125b is enriched in EVs from late-passage iMSCs, while inhibition of miR-125b in iMSCs lead to production of functionally effective EVs in suppressing the expression of Th1 and Th17 cytokines production in splenocytes. MicroRNA-125b is known as a tumor-suppressor miRNA capable of suppressing cell proliferation in neural progenitor cells [44] and several types of cancer cells including medulloblastoma, prostate cancer, colorectal cancer and breast cancer [44-47]. Consistent with these findings, inhibition of miR-125b in late-passage iMSCs significantly increased their proliferation. Similarly, the most abundant miRNA in EVs from late-passage iMSCs is miR-574-3p and its role has been reported to be a tumor suppressor miRNA in various cancers as well [48-50]. Therefore, our results suggest that the increased levels of miR-125b and miR-574-3p in late passage iMSCs may contribute to a decrease in cell proliferation of late-passage iMSCs. However, the roles of miR-125b in immunomodulation are controversial. It has been reported that miR-125b can target key molecules for T cell activation, e.g., BLIMP-1, IL-2Rβ, IL-10Rα, and IFN-γ, contributing to the maintenance of the naïve state in human CD4^+^ T cells [51]. A recent study also demonstrated that exosomal miR-125b derived from MSCs inhibit Th17 cell differentiation by targeting Stat3 [52]. On the contrary, there are reports showing that overexpression of miR-125b induced the activation of macrophages, stimulating T cell activation [53-55]. It is possible that the regulatory mechanism of miR-125b in immunomodulation might change depending on its level and target cells. Furthermore, given the effect of miR-125b on the proliferation of iMSCs, it is also expected that manipulation of individual miRNA in cells could not only change the target miRNA but also subsequently affect characteristics of the cells as well as their EV contents. Therefore, further studies are needed to compare the exact signaling pathways and genes targeted by control EVs vs. manipulated EVs as well as their molecule profiling.

There are still major challenges in developing MSC therapies because of significant variations in the therapeutic efficacy of tissue-derived MSCs due to differences in culture methods, tissue sources and donors. Likewise, EV-based therapies pose the same challenge of functional variation because the contents of MSC-EVs would change as their parent MSCs. Numerous studies have compared the therapeutic potency of MSCs from different tissue sources, but the results have been inconsistent. Also, our previous study [11] and data herein demonstrated that EVs from early-passage MSCs exhibit better immunomodulatory potency, indicating the cellular age of MSCs significantly affects their therapeutic efficacy *in vivo*. Therefore, cellular age should be considered in the production of functionally effective EVs. In this regard, our study has important clinical implications because the use of iPSCs as a starting material to produce MSCs can secure unlimited, reproducible and clinically compliant MSCs and avoid donor-to-donor variations, leading to the development of effective and robust MSC-EV therapies. Furthermore, miR-21 and miR-125b can be used as surrogate markers to validate the immunomodulatory function of EVs as we found that their expression levels in iEVs are correlated to the immune regulatory function of iEVs.

In summary, our comparative analysis on molecular profiles of functionally effective iMSC-EVs identified molecules essential for their immune modulation activity.

## Supplementary Materials

The Supplementary data can be found online at: www.aginganddisease.org/EN/10.14336/AD.2021.0621.


