# Supplementary material for: Identification of Molecules Responsible for Therapeutic Effects of Extracellular Vesicles Produced from iPSC-Derived MSCs on Sjo¨gren’s Syndrome
Source: Aging Dis. 2021 Sep 1;12(6):1409–22. doi: 10.14336/AD.2021.0621 (PMC8407887; doi:10.14336/AD.2021.0621)
Supplement: Supplementary file 1 [file AD-12-6-1409-s.pdf]

SUPPLEMENTARY DATA

Identification of Molecules Responsible for Therapeutic Effects of Extracellular Vesicles Produced from iPSC-Derived MSCs on Sjögren's Syndrome

Hyemee Kim[1#], Qingguo Zhao[1#], Heather Barreda[1], Gagandeep Kaur[1], Bo Hai[1], Jong Min Choi[2], Sung Youn Jung[2], Fei Liu[1,*], Ryang Hwa Lee[1,*]

© 2021. Kim H et al. Published online at http://www.aginganddisease.org/EN/10.14336/AD.2021.0621

SUPPLEMENTARY DATA

Supplementary Table 1. A list of proteins found in P5 EVs and P15 EVs.

Symbol	P5 EVs			P15 EVs		
	SRA*	usPept#	iFOT†	SRA	usPept	iFOT
EIF5A	S	2	946	S	1	30.5
NPM1	S	2	230	S	1	8.3
PPA1	S	2	70.8	S	1	2.9
HSPG2	S	9	13.1	S	1	0.62
ITIH3	S	2	22.3	R	0	1.3
C3	S	1	1.4	S	1	0.09
NME2	S	1	346	S	1	31.7
LOXL2	S	0	0.34	R	0	0.05
RPLP0	S	3	98.9	S	1	15
FERMT2	S	2	35.3	S	1	6.1
UBE2V1	S	0	12.7	S	0	2.9
UBE2V2	S	0	14	S	0	3.2
PAIP2	S	1	77.3	S	1	19.2
MMP12	S	1	5.8	S	1	1.5
EEF2	S	4	279	S	2	72.6
CPE	S	1	15.2	S	2	4
COL12A1	S	13	8.7	S	2	2.3
DSP	S	2	9.3	S	2	2.6
DCTN1	S	1	8.8	S	1	2.5
DYNC1H1	S	5	8.4	S	2	2.4
RAP1A	S	2	313	S	1	96.9
POSTN	S	1	2.8	R	0	0.91
KRT14	S	1	61.7	S	1	20.1
TGFB1	S	1	259	S	1	87.4
KARS	S	1	7.2	R	0	2.5
HIST1H2BP	S	0	56.4	S	0	19.6
HIST1H2BQ	S	0	56.4	S	0	19.6
HIST1H2BR	S	0	56.4	S	0	19.6
HIST3H2BA	S	0	64.4	S	0	22.4
HIST2H2BE	S	0	64.4	S	0	22.4
HIST1H2BC	S	0	64.4	S	0	22.4
HIST1H2BB	S	0	64.4	S	0	22.4
HIST1H2BF	S	0	64.4	S	0	22.4
HIST1H2BG	S	0	64.4	S	0	22.4
HIST1H2BH	S	0	64.4	S	0	22.4
HIST1H2BJ	S	0	64.4	S	0	22.4
HIST1H2BK	S	0	64.4	S	0	22.4
HIST1H2BL	S	0	64.4	S	0	22.4
HIST1H2BM	S	0	64.4	S	0	22.4
HIST1H2BN	S	0	64.4	S	0	22.4
FBN1	S	9	22.6	S	4	8.7
YWHAZ	S	4	5256	S	1	2105
HSPA8	S	3	13.8	S	1	6.3
HSP90AA1	S	5	41.5	S	2	19.6
PLOD1	S	6	381	S	4	185
SPARC	S	4	16351	S	3	8718
SMS	S	3	231	S	2	125
EXT2	S	1	2.4	S	1	1.4

© 2021. Kim H et al. Published online at http://www.aginganddisease.org/EN/10.14336/AD.2021.0621

KRT5	S	1	66.6	S	0	39.1
ATP6V1A	S	2	4.5	A	0	2.7
COL6A2	S	3	25.2	S	2	16.6
LAMA2	S	4	27.3	S	3	19
BGN	S	13	22428	S	13	16649
VCL	S	4	63.3	S	1	47.4
SEPHS1	S	3	107	S	2	82.5
ENO1	S	0	55.9	S	0	43.7
ENO1B	S	0	55.9	S	0	43.7
LAMA4	S	6	63.6	S	3	50.1
USP14	S	2	47.9	S	1	37.9
TFG	S	2	60	S	1	47.6
COL5A1	S	5	153	S	3	123
NF2	S	1	13.5	S	1	11.1
GNB1	S	2	89.1	S	0	74.9
SERPINE1	S	1	73.5	S	1	61.9
CDH11	S	0	7.2	S	0	6.1
CLTC	S	12	36.5	S	4	31
FN1	S	41	1180	S	28	1051
ECE1	R	0	3.3	S	1	3
RAN	S	1	158	S	1	148
TTYH3	S	1	3.2	S	1	3
TARDBP	S	1	94.4	S	1	92.7
OLFML2B	S	5	72.2	S	2	75.9
GSN	S	4	311	S	2	332
IGFBP7	S	1	7.5	S	1	8.1
CLSTN1	S	4	55	S	2	60.8
COL1A1	S	9	97	S	5	111
MMP2	S	8	127	S	2	152
HNRNPA2B1	S	3	18	S	1	22.1
THBS1	S	4	34.7	S	3	46.9
SPTAN1	S	16	82.3	S	8	115
TUBA1A	S	0	78.9	S	0	118
ZYX	S	1	2	R	0	3
LAMB1	S	4	122	S	3	184
TPM2	S	1	13.1	S	3	19.9
HDGF	S	1	14.8	R	0	23.4
PCOLCE	S	1	273	S	2	463
PSMB2	S	2	89.8	S	2	156
AK2	S	1	5.2	S	1	9.1
APP	S	5	342	S	4	626
P4HA2	S	1	0.81	A	0	1.5
PREP	S	1	1	S	1	1.9
COL6A3	S	4	1.4	S	1	2.7
ACTN1	S	12	5643	S	7	10945
PSMA6	S	3	286	S	2	560
CCDC80	S	2	9.7	S	1	19.2
AP2A1	S	0	1.3	R	0	2.7
FLNB	S	3	1.8	S	2	3.8
PRPH	S	3	107	S	3	229
PRDX1	S	1	6.9	S	1	14.8
HAPLN3	S	2	70.4	S	2	152
PAM	S	1	2.4	S	2	5.2
INHBA	S	4	27.5	S	2	60.7
AP2A2	S	0	1.3	R	0	2.9
RFC2	R	0	55.9	S	1	125
HSPA4	S	4	71.9	S	3	163
SNRPG	S	1	61.2	S	1	139
ASNA1	S	1	3	S	1	6.9

TPM1	S	3	344	S	2	807
FSCN1	S	2	41.8	S	2	98.9
GALNT5	S	2	81.2	S	2	195
PDIA4	S	2	6.4	S	1	15.5
KRT77	S	2	25.9	S	2	63.9
FLNA	S	13	242	S	4	600
XYLT2	S	1	6	S	1	14.9
UBA1Y	S	2	7.3	S	1	18.4
NUCB1	S	4	326	S	4	825
LDHB	S	2	167	S	3	427
BMP1	S	3	16.1	S	3	41.6
TPI1	S	2	201	S	2	526
PLOD3	S	2	13.8	S	1	38.2
DPP3	S	4	24.2	S	2	67.9
BIN1	S	2	25.3	S	1	74.4
TAGLN	S	4	199	S	4	596
ARPC1A	S	2	26.5	S	1	81.5
FLNC	S	4	15.4	S	3	47.5
BAG3	R	0	0.81	S	1	2.5
TGFBI	S	3	53.6	S	3	170
USP5	S	1	3.7	S	1	12
RTN4	S	1	2.8	S	1	9.5
AGRN	S	1	11.1	S	1	38.7
LAMC1	S	6	31.2	S	3	114
SF3B3	S	2	1.2	S	1	4.4
BSN	S	1	0.62	R	0	2.3
UBE2L3	S	0	57.1	S	0	213
FSTL1	S	1	70.3	S	1	267
ENO2	S	2	207	S	2	802
ATP6V1B2	S	1	2.6	S	1	10.5
ACTC1	S	1	142	S	2	586
PSMA4	S	2	10	S	2	41.9
SPTBN1	S	7	21.1	S	9	88.5
ARHGAP1	S	1	15.9	S	1	66.9
SEMA7A	S	1	73.7	S	1	311
HAPLN1	S	5	72.3	S	3	322
UGP2	S	2	31.4	S	1	142
FAT1	S	1	0.93	S	1	4.5
ALDOA	S	1	139	S	1	674
WNT5A	S	1	1.6	R	0	7.9
MVP	S	1	0.78	S	1	3.9
DPYSL2	S	0	80.3	S	0	406
PXDN	S	4	10.5	S	4	55.8
CALU	S	4	930	S	4	5080
MYL6	S	3	293	S	2	1607
DPYSL3	S	0	82.9	S	1	459
EFEMP2	S	1	68.1	S	1	381
KRT78	S	1	4.8	S	1	27
PRSS23	S	2	28.9	S	1	166
COL4A1	S	3	123	S	3	721
CDH6	S	2	9.6	S	1	56.5
SPOCK1	S	2	2	S	1	11.9
RPL5	S	1	4.2	S	1	25.9
SEMA5A	S	1	18.1	S	1	112
DNM1L	S	1	0.92	A	0	5.9
ACTB	S	8	1742	S	7	11343
PSMB3	S	1	22.1	S	1	146
EHD2	S	2	16.4	S	2	109
TIMP2	S	2	9.1	S	1	62

© 2021. Kim H et al. Published online at http://www.aginganddisease.org/EN/10.14336/AD.2021.0621

YWHAE	S	3	235	S	2	1654
GDI2	S	3	158	S	2	1314
CTNNB1	S	2	2.1	S	1	18.1
COL4A5	S	1	1.6	S	1	14.1
PRDX6	S	1	35.8	S	1	338
TUBB5	S	1	152	S	1	1469
VCAN	S	6	23.3	S	4	228
ACTN4	S	3	141	S	2	1401
CTPS	S	1	1.6	S	1	16.3
PRSS2	S	1	104	S	1	1118
PKM	S	9	134	S	8	1454
GALNT10	S	1	6.7	S	1	72.8
GLG1	S	1	0.1	S	1	1.1
PLS3	S	3	33.6	S	5	386
SEPT7	S	1	19.1	S	2	224
NID2	S	1	1.2	S	1	15.5
SERPINH1	S	1	5.8	S	3	78
GAPDH	S	0	19.9	S	0	273
GAPDH-PS15	S	0	19.9	S	0	273
PCDH10	A	0	0.16	S	1	2.2
PFKP	R	0	1.1	S	1	16.6
ACLY	S	1	1.5	S	1	22.8
HSP90AB1	S	4	19.7	S	1	301
ACTR2	S	1	4.3	R	0	66.9
ACTR3	S	3	23.6	S	2	371
EFNB2	R	0	7	S	1	145
ALCAM	S	2	23.8	S	2	502
SERPINE2	S	0	3.2	S	1	68
HSPA5	R	0	0.27	S	1	5.8
GFRA1	S	1	0.6	S	1	13.6
PUF60	S	1	0.53	S	1	12.5
TXNDC5	R	0	41.8	S	2	1065
CALM3	S	2	49.8	S	2	1316
SEPT2	S	3	22.6	S	3	627
MYL12B	S	2	39	S	2	1083
EHD1	R	0	3.2	S	3	92.3
MEGF8	S	1	0.71	S	2	21.4
GANAB	S	1	1.1	S	2	34.8
HYOU1	S	1	1.8	S	3	70.4
EIF4A2	S	0	0.82	S	0	33.3
EIF4A1	S	0	0.74	S	0	30.1
TRY4	R	0	2.9	R	0	128
GM5771	R	0	2.9	R	0	128
PRSS1	R	0	3.1	R	0	139
TLN1	S	1	2.1	S	7	99.6
COPS5	R	0	0.49	S	1	24.3
ENPP2	R	0	0.59	S	2	29.3
PPP2R1A	S	0	0.47	S	0	25
PPP2R1B	S	0	0.46	S	0	24.6
ANXA2	S	1	17.1	S	2	1111
LTBP1	S	1	3.8	S	1	285
ACAN	S	1	1.3	S	1	179
HSPH1	S	1	0.1	S	1	13.8
PYGB	S	1	0.23	S	2	32.7
SYNCRIP	S	1	0.51	S	1	75.5
CAPZA2	R	0	0.22	S	1	87.1
HBB-BS		0	0	S	0	6.6
HBB-B1		0	0	S	0	6.3
HBB-B2		0	0	S	0	6.3

© 2021. Kim H et al. Published online at http://www.aginganddisease.org/EN/10.14336/AD.2021.0621

UBA52		0	0	R	0	0.92
KRT73	A	0	0	S	0	120
UBB		0	0	R	0	0.33
UBC		0	0	R	0	0.14
PRDX4		0	0	S	1	190
RPS24		0	0	S	1	21.2
KRT6A	A	0	0	S	0	41.7
KRT75	A	0	0	S	0	39.1
LMNA		0	0	S	1	0.66
SRPX2		0	0	S	1	13.7
PDCD6IP		0	0	S	2	49.8
RPS2		0	0	S	1	8.4
RRAS		0	0	S	1	5.4
CDH13		0	0	R	0	5.5
PABPC4		0	0	S	0	26.5
PABPC1		0	0	S	0	24.7
IQGAP1		0	0	S	2	42.9
ACTR1B		0	0	S	0	18.9
ACTR1A		0	0	S	0	18
TUBB2B	A	0	0	S	0	707
ATP5B		0	0	S	1	9
HSPD1		0	0	R	0	17.4
RPS10		0	0	S	1	23
CAMK2D		0	0	S	1	6.1
ESD		0	0	S	1	73.9
YWHAB	A	0	0	S	1	872
ERO1L		0	0	S	1	5.8
GM10705	A	0	0	S	0	213
ECM2		0	0	S	1	11.1
RAB2A		0	0	S	1	11.1
PAFAH1B1		0	0	S	1	9.2
STAT1		0	0	R	0	12.2
NPEPPS		0	0	S	2	28.3
EPPK1	S	1	0	S	1	7.8
PPP2R2A		0	0	S	0	3.8
PDCD6		0	0	S	1	31.1
YWHAQ	A	0	0	S	1	373
ADAMTS7		0	0	S	1	21
NEO1		0	0	S	1	14
GNAI2		0	0	S	2	26.7
DDB1	R	0	0	S	1	4.6
ASNS		0	0	S	1	33.5
TUBB2A	A	0	0	S	0	707
PPP2R2B		0	0	S	0	4
ITGA3		0	0	S	1	6.8
LOXL3	S	1	0	S	2	12
MYL9	R	0	0	S	1	134
EPHA5	A	0	0	S	2	255
RNASE4	A	0	0	S	1	18.1
TPM3	A	0	0	S	0	1.5
CAPN2		0	0	S	1	30.9
LAP3		0	0	S	1	21.3
PTPRK		0	0	S	1	0.05
VPS35		0	0	S	1	5.4
WARS		0	0	R	0	3.9
ATRN		0	0	R	0	7
CAPG		0	0	S	1	18.7
IDS		0	0	S	1	20.2
NPR3		0	0	S	1	76.9

© 2021. Kim H et al. Published online at http://www.aginganddisease.org/EN/10.14336/AD.2021.0621

PVRL2		0	0	S	1	52.5
FBLN5		0	0	S	1	56
CUL1		0	0	S	1	1.5
LSM2		0	0	S	1	14
SNX3		0	0	S	1	9.5
MMP19		0	0	S	1	1.3
SF3B6		0	0	S	1	17.4
RGMB		0	0	S	1	54.7
GALNT14		0	0	S	1	61
CPA4		0	0	S	1	33
ATP6V1D		0	0	S	1	8.8
HTRA3		0	0	S	1	5.5
SIL1		0	0	S	1	14
ATP6V1H		0	0	S	1	10.8
TSPAN9		0	0	S	1	30.8
DCUN1D1		0	0	S	1	6.9
KIRREL		0	0	S	1	7.1
MAN1B1		0	0	S	1	2.2
CKB		0	0	S	1	82.3
CDH8		0	0	S	1	8.3
MGAT1		0	0	S	1	32.6
PCSK5		0	0	S	1	1.7
MPRIP		0	0	S	1	0.96
TXNL1		0	0	S	1	10.1
CARM1		0	0	S	1	19.3
ASF1A		0	0	S	1	41.4
MVB12B		0	0	S	1	2.4
CANT1		0	0	S	1	7.3
TRIM23		0	0	S	1	41
REXO2		0	0	S	1	8
RCC2		0	0	S	1	1.8
OLFM2		0	0	S	1	6.8
ROCK1		0	0	S	0	0.29
ROCK2		0	0	S	0	0.31
4732456N10RIK	S	1	194	A	0	0
TSPAN4	S	1	179		0	0
TSN	S	1	160		0	0
ITGB1	S	2	158		0	0
TUBA1C	S	1	119	A	0	0
ASPM	S	1	111		0	0
TUBA1B	S	0	109	A	0	0
CRK	S	1	96.5		0	0
RBBP4	S	1	82.8		0	0
GNB2	S	3	78.1	A	0	0
TUBB4B	S	1	58.9	A	0	0
EEF1G	S	1	51		0	0
TGFB2	S	2	39.7		0	0
CDC37	S	1	38.1		0	0
PFDN5	S	1	36.1		0	0
CPB2	S	1	34.9		0	0
NAP1L1	S	1	32.8		0	0
SERPINI2	S	1	30.6	S	1	0
PCBD1	S	1	29.4		0	0
RPLP2	S	2	27.4	S	1	0
ABI3BP	S	1	26.9	R	0	0
PGD	S	1	26.7		0	0
PSMC1	S	3	25.2		0	0
EIF3A	S	2	23.8		0	0
IPO5	S	4	22.8	A	0	0

© 2021. Kim H et al. Published online at http://www.aginganddisease.org/EN/10.14336/AD.2021.0621

SUPPLEMENTARY DATA

PARVA	S	1	21.5		0	0
2210010C04RIK	S	1	20.5		0	0
TWF2	S	1	20.1	R	0	0
ARPC4	S	1	19.3		0	0
RPS4X	S	1	18.8		0	0
HNRNPK	S	1	15.6		0	0
RANBP1	S	1	15.1		0	0
JAG1	S	1	15.1		0	0
LMAN2	S	1	15		0	0
PAICS	S	1	14.6		0	0
NUTF2	R	0	13.8	S	1	0
OTUB1	S	1	13.8		0	0
TARS	S	1	13.1		0	0
CHID1	S	1	12.4		0	0
CAPRIN1	S	1	12.3		0	0
PFAS	S	1	11.7		0	0
COPS6	S	1	11.5		0	0
XPO1	S	2	11.3		0	0
THBS2	S	1	10.6	A	0	0
COPS2	S	1	10.1		0	0
FAM20B	S	1	9.9		0	0
SSB	S	2	9	R	0	0
DLG1	S	1	7.9		0	0
ALDH16A1	S	1	7.9		0	0
CBX1	S	2	7.7		0	0
TINAGL1	S	1	7.4		0	0
COPS7B	S	1	7.1		0	0
NMT1	S	1	7		0	0
MAMDC2	S	2	6.6		0	0
GMPR2	S	1	6.3		0	0
AP2B1	S	0	6.1		0	0
P4HB	S	2	6		0	0
LAMA5	S	1	6		0	0
GNB4	S	0	5.7		0	0
PRPSAP2	S	1	5.7		0	0
IDH1	S	1	5.7		0	0
HNRNPA1	S	1	5.5		0	0
ANGPTL2	S	1	5.3		0	0
CDC42	S	1	5.1		0	0
ASPH	S	1	5.1		0	0
PSMA8	A	0	4.9		0	0
LDLR	S	1	4.7		0	0
SKP1A	S	1	4.6		0	0
EIF3B	S	2	4.5		0	0
PRPF19	S	1	4.3		0	0
EVA1B	S	1	4.2		0	0
SERPINC1	S	2	4.1		0	0
COPB1	S	2	4.1		0	0
OS9	S	1	4		0	0
EFTUD2	S	1	3.9		0	0
B4GAT1	S	1	3.6		0	0
H2-K1	S	1	3.4		0	0
ALDOART1	S	1	3.4	A	0	0
FBN2	S	3	3.3		0	0
CACYBP	A	0	3.2		0	0
SERPINB3C	S	0	3	A	0	0
COPS4	S	1	3		0	0
CCT7	S	2	2.9		0	0
HNRNPC	S	2	2.8		0	0

SEPT11	S	1	2.8		0	0
CEMIP	S	1	2.7		0	0
CUL4A	S	1	2.7		0	0
CSNK2A1	S	1	2.6		0	0
LTA4H	S	1	2.5		0	0
LTBP3	S	1	2.4	R	0	0
IARS	S	1	2.3		0	0
MYH10	S	0	2.2		0	0
CDH2	S	1	2.2		0	0
PBDC1	S	1	2.1		0	0
CS	R	0	1.9		0	0
CFL2	S	1	1.8		0	0
NRP1	S	2	1.7		0	0
SEC24A	S	1	1.7		0	0
PABPN1	S	1	1.4		0	0
PPME1	S	1	1.4		0	0
RAB1B	S	1	1.3	S	1	0
TMPO	R	0	1.2		0	0
CAND1	S	1	1.1	A	0	0
SND1	S	1	1		0	0
COPS3	S	1	0.95		0	0
KANK2	S	1	0.95		0	0
RPS6	A	0	0.85		0	0
COPG1	R	0	0.79		0	0
PRPF8	S	2	0.78		0	0
TENM3	S	1	0.77		0	0
EPRS	R	0	0.75	S	1	0
DCPS	S	1	0.71		0	0
UBXN7	S	1	0.69		0	0
EIF3D	R	0	0.64		0	0
A2M	S	1	0.63		0	0
EMILIN1	S	1	0.62		0	0
SSC5D	S	2	0.62		0	0
HIST1H4H	S	0	0.59		0	0
HIST2H4	S	0	0.59		0	0
HIST1H4C	S	0	0.59		0	0
HIST1H4D	S	0	0.59		0	0
HIST1H4I	S	0	0.59		0	0
HIST1H4J	S	0	0.59		0	0
HIST1H4K	S	0	0.59		0	0
HIST1H4N	S	0	0.59		0	0
HIST4H4	S	0	0.59		0	0
HIST1H4A	S	0	0.59		0	0
HIST1H4B	S	0	0.59		0	0
HIST1H4M	S	0	0.59		0	0
MIF	A	0	0.59		0	0
GSPT1	S	1	0.58		0	0
EIF4G3	S	1	0.54		0	0
KCNH5	R	0	0.51		0	0
DAG1	S	1	0.34		0	0
MYOF	S	1	0.28		0	0
DDX42	S	1	0.27		0	0
GHRL	R	0	0.24		0	0
GFPT1	S	1	0.23		0	0
PCBP1	S	1	0.19		0	0
VDAC1	S	1	0.17		0	0
LDHA	R	0	0.14		0	0
EML1	S	0	0.14		0	0
EML4	S	0	0.13		0	0

© 2021. Kim H et al. Published online at http://www.aginganddisease.org/EN/10.14336/AD.2021.0621

FAP	A	0	0.12		0	0
EIF2S3Y	S	0	0.11		0	0
EIF3M	A	0	0.11		0	0
EIF2S3X	S	0	0.11		0	0
SNRNP200	R	0	0.1		0	0
KRT10	S	1	0.08	R	0	0
TNC	S	1	0.04		0	0
LTBP2	R	0	0.02		0	0
NUMA1	R	0	0.01		0	0

*Strict, Relaxed, and All (SRA)
#Proteins were assembled with unique and strict peptides (<1% FDR, usPepts)
†For label-free quantification fraction of total (iFOT)

Supplementary Table 2. IPA analyses on proteins completely unique to P5 EVs with P value < 0.05.

Ingenuity Canonical Pathways	-log(p-value)	Ratio	z-score	Molecules
Germ Cell-Sertoli Cell Junction Signaling	6.13	0.0526	#NUM!	A2M,Cdc42,CDH2,CFL2,ITGB1,TGFB2,TUBA1B,TUBA1C,TUBB4B
Regulation of eIF4 and p70S6K Signaling	5.96	0.0503	#NUM!	EIF2S3,EIF3A,EIF3B,EIF3D,EIF3M,EIF4G3,ITGB1,RPS4Y1,RPS6
Epithelial Adherens Junction Signaling	5.38	0.0506	#NUM!	ARPC4,CDH2,CRK,MYH10,TGFB2,TUBA1B,TUBA1C,TUBB4B
Coronavirus Replication Pathway	5.2	0.111	#NUM!	COPB1,COPG1,TUBA1B,TUBA1C,TUBB4B
EIF2 Signaling	5.16	0.0402	#NUM!	EIF2S3,EIF3A,EIF3B,EIF3D,EIF3M,EIF4G3,RPLP2,RPS4Y1,RPS6
NER (Nucleotide Excision Repair, Enhanced Pathway)	4.5	0.0583	#NUM!	Cops2,COPS3,COPS4,COPS6,COPS7B,CUL4A
mTOR Signaling	4.45	0.0377	#NUM!	Cdc42,EIF3A,EIF3B,EIF3D,EIF3M,EIF4G3,RPS4Y1,RPS6
RhoGDI Signaling	4.41	0.0372	#NUM!	ARPC4,Cdc42,CDH2,CFL2,GNB2,GNB4,ITGB1,MYH10
RAN Signaling	3.91	0.176	#NUM!	IPO5,RANBP1,XPO1
Semaphorin Signaling in Neurons	3.33	0.0645	#NUM!	Cdc42,CFL2,ITGB1,NRP1
Remodeling of Epithelial Adherens Junctions	3.17	0.0588	#NUM!	ARPC4,TUBA1B,TUBA1C,TUBB4B
Axonal Guidance Signaling	3.11	0.0198	#NUM!	ARPC4,CFL2,CRK,GNB2,GNB4,ITGB1,NRP1,TUBA1B,TUBA1C,TUBB4B
Ephrin B Signaling	3.08	0.0556	#NUM!	CFL2,GNB2,GNB4,HNRNPK
Caveolar-mediated Endocytosis Signaling	3.01	0.0533	#NUM!	COPB1,COPG1,HLA-A,ITGB1
Signaling by Rho Family GTPases	3	0.0261	#NUM!	ARPC4,Cdc42,CDH2,CFL2,GNB2,GNB4,ITGB1
Ephrin Receptor Signaling	2.93	0.0299	#NUM!	ARPC4,CFL2,CRK,GNB2,GNB4,ITGB1
Sertoli Cell-Sertoli Cell Junction Signaling	2.88	0.0291	#NUM!	A2M,DLG1,ITGB1,TUBA1B,TUBA1C,TUBB4B
tRNA Charging	2.82	0.0769	#NUM!	EPRS1,IARS1,TARS1
Integrin Signaling	2.8	0.0282	#NUM!	ARPC4,Cdc42,CRK,ITGB1,PARVA,TSPAN4
Purine Nucleotides De Novo Biosynthesis II	2.75	0.182	#NUM!	PAICS,PFAS

SUPPLEMENTARY DATA

Spliceosomal Cycle	2.53	0.0612	#NUM!	EFTUD2,PRPF19,SNRNP200
ILK Signaling	2.21	0.0253	#NUM!	Cdc42,CFL2,ITGB1,MYH10,PARVA
Reelin Signaling in Neurons	2.2	0.0317	#NUM!	ARPC4,CDH2,CRK,ITGB1
Phagosome Maturation	1.85	0.0252	#NUM!	HLA-A,TUBA1B,TUBA1C,TUBB4B
Actin Cytoskeleton Signaling	1.84	0.0204	#NUM!	ARPC4,CFL2,CRK,ITGB1,MYH10
CXCR4 Signaling	1.78	0.024	#NUM!	Cdc42,CRK,GNB2,GNB4
Actin Nucleation by ARP-WASP Complex	1.76	0.0323	#NUM!	ARPC4,Cdc42,ITGB1
5-aminoimidazole Ribonucleotide Biosynthesis I	1.76	0.333	#NUM!	PFAS
Inosine-5'-phosphate Biosynthesis II	1.76	0.333	#NUM!	PAICS
Tyrosine Biosynthesis IV	1.76	0.333	#NUM!	PCBD1
Coagulation System	1.75	0.0571	#NUM!	A2M,SERPINC1
Inhibition of Matrix Metalloproteases	1.66	0.0513	#NUM!	A2M,THBS2
Pentose Phosphate Pathway (Oxidative Branch)	1.64	0.25	#NUM!	PGD
Phenylalanine Degradation I (Aerobic)	1.64	0.25	#NUM!	PCBD1
PD-1, PD-L1 cancer immunotherapy pathway	1.62	0.0283	#NUM!	CSNK2A1,HLA-A,TGFB2
Paxillin Signaling	1.6	0.0278	#NUM!	CRK,ITGB1,PARVA
Clathrin-mediated Endocytosis Signaling	1.58	0.0207	#NUM!	ARPC4,CSNK2A1,ITGB1,LDLR
G Protein Signaling Mediated by Tubby	1.57	0.0455	#NUM!	GNB2,GNB4
Eumelanin Biosynthesis	1.54	0.2	#NUM!	MIF
Regulation of Actin-based Motility by Rho	1.52	0.0259	#NUM!	ARPC4,Cdc42,ITGB1
Role of Tissue Factor in Cancer	1.52	0.0259	#NUM!	CFL2,ITGB1,P4HB
Neuregulin Signaling	1.51	0.0256	#NUM!	CRK,ITGB1,RPS6
Apelin Muscle Signaling Pathway	1.5	0.0417	#NUM!	GNB2,GNB4
Pyruvate Fermentation to Lactate	1.46	0.167	#NUM!	LDHA
UDP-N-acetyl-D-glucosamine Biosynthesis II	1.46	0.167	#NUM!	GFPT1
Synaptogenesis Signaling Pathway	1.44	0.016	#NUM!	ARPC4,CDH2,CRK,NAP1L1,THBS2
14-3-3-mediated Signaling	1.42	0.0236	#NUM!	TUBA1B,TUBA1C,TUBB4B
fMLP Signaling in Neutrophils	1.38	0.0229	#NUM!	ARPC4,GNB2,GNB4
FAT10 Signaling Pathway	1.37	0.0357	#NUM!	PSMA8,PSMC1

© 2021. Kim H et al. Published online at http://www.aginganddisease.org/EN/10.14336/AD.2021.0621

CCR3 Signaling in Eosinophils	1.35	0.0222	#NUM!	CFL2,GNB2,GNB4
Molecular Mechanisms of Cancer	1.33	0.0135	#NUM!	Cdc42,CRK,GNB2,GNB4,ITGB1,TGFB2
Rac Signaling	1.33	0.0217	#NUM!	ARPC4,CFL2,ITGB1
SPINK1 Pancreatic Cancer Pathway	1.32	0.0333	#NUM!	2210010C04Rik,CPB2
Polyamine Regulation in Colon Cancer	1.29	0.0323	#NUM!	PSMA8,PSMC1
Pentose Phosphate Pathway	1.25	0.1	#NUM!	PGD
Glutamate Receptor Signaling	1.25	0.0303	#NUM!	GNB2,GNB4
Semaphorin Neuronal Repulsive Signaling Pathway	1.23	0.0199	#NUM!	CFL2,ITGB1,NRP1
Agrin Interactions at Neuromuscular Junction	1.2	0.0286	#NUM!	DAG1,ITGB1
Cardiac Hypertrophy Signaling	1.19	0.0155	#NUM!	Cdc42,GNB2,GNB4,TGFB2
Cleavage and Polyadenylation of Pre-mRNA	1.17	0.0833	#NUM!	PABPN1
Inhibition of ARE-Mediated mRNA Degradation Pathway	1.17	0.0186	#NUM!	PABPN1,PSMA8,PSMC1
Hypoxia Signaling in the Cardiovascular System	1.16	0.027	#NUM!	LDHA,P4HB
Colorectal Cancer Metastasis Signaling	1.14	0.0149	#NUM!	Cdc42,GNB2,GNB4,TGFB2
Human Embryonic Stem Cell Pluripotency	1.14	0.0181	#NUM!	GNB2,GNB4,TGFB2
Antiproliferative Role of Somatostatin Receptor 2	1.13	0.026	#NUM!	GNB2,GNB4
VDR/RXR Activation	1.12	0.0256	#NUM!	CSNK2A1,TGFB2
Gαq Signaling	1.11	0.0176	#NUM!	Cdc42,GNB2,GNB4
Leukotriene Biosynthesis	1.1	0.0714	#NUM!	LTA4H
Wnt/β-catenin Signaling	1.09	0.0173	#NUM!	CDH2,CSNK2A1,TGFB2
Huntington's Disease Signaling	1.08	0.0142	#NUM!	GNB2,GNB4,PSMA8,PSMC1
BAG2 Signaling Pathway	1.06	0.0238	#NUM!	PSMA8,PSMC1
Tumor Microenvironment Pathway	1.06	0.0168	#NUM!	HLA-A,TGFB2,TNC
Extrinsic Prothrombin Activation Pathway	1.05	0.0625	#NUM!	SERPINC1
Chondroitin Sulfate Degradation (Metazoa)	1.05	0.0625	#NUM!	CEMIP
PDGF Signaling	1.05	0.0233	#NUM!	CRK,CSNK2A1
Sirtuin Signaling Pathway	1.04	0.0137	#NUM!	LDHA,TUBA1B,TUBA1C,VDAC1
Dermatan Sulfate Degradation (Metazoa)	1.02	0.0588	#NUM!	CEMIP
Cardiac Hypertrophy Signaling (Enhanced)	1.02	0.0112	#NUM!	DLG1,GNB2,GNB4,ITGB1,RPS6,TGFB2

© 2021. Kim H et al. Published online at http://www.aginganddisease.org/EN/10.14336/AD.2021.0621

Unfolded protein response	1.01	0.0222	#NUM!	OS9,P4HB
Fcγ Receptor-mediated Phagocytosis in Macrophages and Monocytes	0.983	0.0213	#NUM!	ARPC4,CRK
IL-1 Signaling	0.983	0.0213	#NUM!	GNB2,GNB4
Hepatic Fibrosis / Hepatic Stellate Cell Activation	0.979	0.0155	#NUM!	A2M,MYH10,TGFB2
Regulation of the Epithelial-Mesenchymal Transition Pathway	0.975	0.0154	#NUM!	CDH2,JAG1,TGFB2
HER-2 Signaling in Breast Cancer	0.971	0.0153	#NUM!	COPS6,ITGB1,RPS6
Gap Junction Signaling	0.963	0.0152	#NUM!	TUBA1B,TUBA1C,TUBB4B
Natural Killer Cell Signaling	0.955	0.0151	#NUM!	CFL2,HLA-A,ITGB1
HIF1α Signaling	0.914	0.0144	#NUM!	LDHA,RPS6,TGFB2
Virus Entry via Endocytic Pathways	0.91	0.0192	#NUM!	HLA-A,ITGB1
Thrombin Signaling	0.907	0.0143	#NUM!	Cdc42,GNB2,GNB4
IL-8 Signaling	0.9	0.0142	#NUM!	Cdc42,GNB2,GNB4
Apelin Cardiac Fibroblast Signaling Pathway	0.9	0.0435	#NUM!	TGFB2
α-Adrenergic Signaling	0.896	0.0189	#NUM!	GNB2,GNB4
Chronic Myeloid Leukemia Signaling	0.889	0.0187	#NUM!	CRK,TGFB2
TCA Cycle II (Eukaryotic)	0.883	0.0417	#NUM!	CS
Breast Cancer Regulation by Stathmin1	0.876	0.0101	#NUM!	GNB2,GNB4,TGFB2,TUBA1B,TUBA1C,TUBB4B
Role of NFAT in Cardiac Hypertrophy	0.863	0.0136	#NUM!	GNB2,GNB4,TGFB2
Systemic Lupus Erythematosus Signaling	0.86	0.01	#NUM!	EFTUD2,HLA-A,HNRNPC,PRPF19,PRPF8,SNRNP200
CDK5 Signaling	0.857	0.0179	#NUM!	ITGB1,LAMA5
FAK Signaling	0.827	0.0171	#NUM!	CRK,ITGB1
PAK Signaling	0.821	0.0169	#NUM!	CFL2,ITGB1
Neuroprotective Role of THOP1 in Alzheimer's Disease	0.821	0.0169	#NUM!	FAP,HLA-A
Gαs Signaling	0.804	0.0165	#NUM!	GNB2,GNB4
Endocannabinoid Developing Neuron Pathway	0.793	0.0163	#NUM!	GNB2,GNB4
RhoA Signaling	0.788	0.0161	#NUM!	ARPC4,CFL2
Airway Inflammation in Asthma	0.767	0.0312	#NUM!	TGFB2
IL-6 Signaling	0.767	0.0156	#NUM!	A2M,CSNK2A1

© 2021. Kim H et al. Published online at http://www.aginganddisease.org/EN/10.14336/AD.2021.0621

SUPPLEMENTARY DATA

G Beta Gamma Signaling	0.762	0.0155	#NUM !	GNB2,GNB4
P2Y Purigenic Receptor Signaling Pathway	0.762	0.0155	#NUM !	GNB2,GNB4
GABA Receptor Signaling	0.75	0.0153	#NUM !	GNB2,GNB4
DNA Methylation and Transcriptional Repression Signaling	0.733	0.0286	#NUM !	RBBP4
MIF-mediated Glucocorticoid Regulation	0.721	0.0278	#NUM !	MIF
Th2 Pathway	0.721	0.0146	#NUM !	HLA-A,JAG1
Gαi Signaling	0.717	0.0145	#NUM !	GNB2,GNB4
Apelin Endothelial Signaling Pathway	0.712	0.0144	#NUM !	GNB2,GNB4
Notch Signaling	0.701	0.0263	#NUM !	JAG1
Thyroid Hormone Metabolism II (via Conjugation and/or Degradation)	0.69	0.0256	#NUM !	B4GAT1
Antigen Presentation Pathway	0.69	0.0256	#NUM !	HLA-A
Protein Kinase A Signaling	0.682	0.0099 5	#NUM !	GNB2,GNB4,MYH10,TGFB2
Endocannabinoid Neuronal Synapse Pathway	0.676	0.0136	#NUM !	GNB2,GNB4
Mechanisms of Viral Exit from Host Cells	0.672	0.0244	#NUM !	XPO1
Phagosome Formation	0.666	0.0134	#NUM !	Cdc42,ITGB1
Intrinsic Prothrombin Activation Pathway	0.662	0.0238	#NUM !	SERPINC1
PTEN Signaling	0.662	0.0133	#NUM !	CSNK2A1,ITGB1
NAD Signaling Pathway	0.658	0.0132	#NUM !	LDHA,TGFB2
B Cell Development	0.654	0.0233	#NUM !	HLA-A
Serotonin Receptor Signaling	0.654	0.0233	#NUM !	PCBD1
Relaxin Signaling	0.65	0.0131	#NUM !	GNB2,GNB4
MIF Regulation of Innate Immunity	0.644	0.0227	#NUM !	MIF
Role of NFAT in Regulation of the Immune Response	0.627	0.0088 2	#NUM !	GNB2,GNB4,HLA-A,IPO5,XPO1
PFKFB4 Signaling Pathway	0.627	0.0217	#NUM !	TGFB2
Aryl Hydrocarbon Receptor Signaling	0.625	0.0126	#NUM !	ALDH16A1,TGFB2
Ephrin A Signaling	0.62	0.0213	#NUM !	CFL2
nNOS Signaling in Skeletal Muscle Cells	0.611	0.0208	#NUM !	DAG1

© 2021. Kim H et al. Published online at http://www.aginganddisease.org/EN/10.14336/AD.2021.0621

SUPPLEMENTARY DATA

HMGB1 Signaling	0.595	0.012	#NUM!	Cdc42,TGFB2
Gustation Pathway	0.595	0.012	#NUM!	GNB2,GNB4
FAT10 Cancer Signaling Pathway	0.595	0.02	#NUM!	TGFB2
Triacylglycerol Degradation	0.588	0.0196	#NUM!	PPME1
Amyloid Processing	0.588	0.0196	#NUM!	CSNK2A1
Androgen Signaling	0.587	0.0118	#NUM!	GNB2,GNB4
Th1 and Th2 Activation Pathway	0.577	0.0116	#NUM!	HLA-A,JAG1
Cardiac β-adrenergic Signaling	0.57	0.0115	#NUM!	GNB2,GNB4
EGF Signaling	0.561	0.0182	#NUM!	CSNK2A1
Tight Junction Signaling	0.556	0.0112	#NUM!	MYH10,TGFB2
Nicotine Degradation III	0.541	0.0172	#NUM!	B4GAT1
Acute Phase Response Signaling	0.533	0.0108	#NUM!	A2M,HNRNPK
GNRH Signaling	0.523	0.0106	#NUM!	GNB2,GNB4
Melatonin Degradation I	0.523	0.0164	#NUM!	B4GAT1
IL-2 Signaling	0.523	0.0164	#NUM!	CSNK2A1
SAPK/JNK Signaling	0.513	0.00833	#NUM!	CRK,GNB2,GNB4,HNRNPK
Regulation Of The Epithelial Mesenchymal Transition By Growth Factors Pathway	0.511	0.0104	#NUM!	CDH2,TGFB2
Leukocyte Extravasation Signaling	0.509	0.0104	#NUM!	CRK,ITGB1
PPARα/RXRα Activation	0.509	0.0104	#NUM!	CAND1,TGFB2
RAR Activation	0.499	0.0102	#NUM!	CSNK2A1,TGFB2
Role of PI3K/AKT Signaling in the Pathogenesis of Influenza	0.495	0.0152	#NUM!	CRK
Pyridoxal 5'-phosphate Salvage Pathway	0.495	0.0152	#NUM!	FAM20B
Nicotine Degradation II	0.495	0.0152	#NUM!	B4GAT1
Superpathway of Melatonin Degradation	0.495	0.0152	#NUM!	B4GAT1
PI3K/AKT Signaling	0.491	0.0101	#NUM!	CDC37,ITGB1
Eicosanoid Signaling	0.489	0.0149	#NUM!	LTA4H
Serotonin Degradation	0.484	0.0147	#NUM!	B4GAT1
Cell Cycle: G1/S Checkpoint Regulation	0.484	0.0147	#NUM!	TGFB2
SPINK1 General Cancer Pathway	0.479	0.0145	#NUM!	2210010C04Rik

SUPPLEMENTARY DATA

Coronavirus Pathogenesis Pathway	0.479	0.00985	#NUM!	RPS4Y1,RPS6
Growth Hormone Signaling	0.469	0.0141	#NUM!	A2M
Glioma Invasiveness Signaling	0.458	0.0137	#NUM!	Cdc42
Heparan Sulfate Biosynthesis (Late Stages)	0.458	0.0137	#NUM!	PPME1
Leptin Signaling in Obesity	0.453	0.0135	#NUM!	GHRL
Agranulocyte Adhesion and Diapedesis	0.45	0.00935	#NUM!	ITGB1,MYH10
ERK/MAPK Signaling	0.45	0.00935	#NUM!	CRK,ITGB1
Macropinocytosis Signaling	0.445	0.0132	#NUM!	ITGB1
Calcium Signaling	0.445	0.00926	#NUM!	ASPH,MYH10
TREM1 Signaling	0.44	0.013	#NUM!	ITGB1
Angiopoietin Signaling	0.44	0.013	#NUM!	CRK
Dopamine Receptor Signaling	0.44	0.013	#NUM!	PCBD1
NF-κB Activation by Viruses	0.435	0.0128	#NUM!	ITGB1
Renal Cell Carcinoma Signaling	0.427	0.0125	#NUM!	CRK
Heparan Sulfate Biosynthesis	0.427	0.0125	#NUM!	PPME1
BEX2 Signaling Pathway	0.423	0.0123	#NUM!	CDH2
TR/RXR Activation	0.411	0.0119	#NUM!	LDLR
Cyclins and Cell Cycle Regulation	0.411	0.0119	#NUM!	TGFB2
VEGF Family Ligand-Receptor Interactions	0.411	0.0119	#NUM!	NRP1
FGF Signaling	0.411	0.0119	#NUM!	CRK
HIPPO signaling	0.407	0.0118	#NUM!	DLG1
Osteoarthritis Pathway	0.401	0.00855	#NUM!	ITGB1,JAG1
Xenobiotic Metabolism AHR Signaling Pathway	0.399	0.0115	#NUM!	ALDH16A1
Regulation Of The Epithelial Mesenchymal Transition In Development Pathway	0.399	0.0115	#NUM!	JAG1
Cdc42 Signaling	0.395	0.00723	#NUM!	ARPC4,CFL2,HLA-A,ITGB1
Regulation of Cellular Mechanics by Calpain Protease	0.391	0.0112	#NUM!	ITGB1
Tec Kinase Signaling	0.39	0.00719	#NUM!	Cdc42,GNB2,GNB4,ITGB1
Acute Myeloid Leukemia Signaling	0.384	0.011	#NUM!	IDH1

© 2021. Kim H et al. Published online at http://www.aginganddisease.org/EN/10.14336/AD.2021.0621

SUPPLEMENTARY DATA

Crosstalk between Dendritic Cells and Natural Killer Cells	0.384	0.011	#NUM!	HLA-A
AMPK Signaling	0.384	0.00826	#NUM!	GNB2,GNB4
IL-4 Signaling	0.38	0.0109	#NUM!	HLA-A
Estrogen Receptor Signaling	0.378	0.00743	#NUM!	CFL2,GNB2,GNB4
Melanocyte Development and Pigmentation Signaling	0.374	0.0106	#NUM!	CRK
TGF-β Signaling	0.367	0.0104	#NUM!	TGFB2
LPS/IL-1 Mediated Inhibition of RXR Function	0.364	0.00794	#NUM!	ALDH16A1,XPO1
ATM Signaling	0.364	0.0103	#NUM!	CBX1
Salvage Pathways of Pyrimidine Ribonucleotides	0.36	0.0102	#NUM!	FAM20B
Sperm Motility	0.36	0.00787	#NUM!	GNB2,GNB4
VEGF Signaling	0.357	0.0101	#NUM!	EIF2S3
Hepatic Fibrosis Signaling Pathway	0.355	0.00716	#NUM!	Cdc42,ITGB1,TGFB2
Sumoylation Pathway	0.344	0.00971	#NUM!	Cdc42
IGF-1 Signaling	0.341	0.00962	#NUM!	CSNK2A1
Insulin Secretion Signaling Pathway	0.333	0.00746	#NUM!	EIF2S3,EIF4G3
Protein Ubiquitination Pathway	0.321	0.00727	#NUM!	HLA-A,PSMC1
Opioid Signaling Pathway	0.32	0.00725	#NUM!	GNB2,GNB4
Airway Pathology in Chronic Obstructive Pulmonary Disease	0.302	0.00847	#NUM!	TGFB2
Sphingosine-1-phosphate Signaling	0.302	0.00847	#NUM!	Cdc42
NGF Signaling	0.302	0.00847	#NUM!	CRK
p38 MAPK Signaling	0.302	0.00847	#NUM!	TGFB2
Cholecystokinin/Gastrin-mediated Signaling	0.299	0.0084	#NUM!	Cdc42
Th1 Pathway	0.292	0.0082	#NUM!	HLA-A
LXR/RXR Activation	0.289	0.00813	#NUM!	LDLR
Glioma Signaling	0.287	0.00806	#NUM!	IDH1
Pancreatic Adenocarcinoma Signaling	0.282	0.00794	#NUM!	TGFB2
GP6 Signaling Pathway	0.28	0.00787	#NUM!	LAMA5

© 2021. Kim H et al. Published online at http://www.aginganddisease.org/EN/10.14336/AD.2021.0621

Phospholipase C Signaling	0.277	0.00613	#NUM!	Cdc42,GNB2,GNB4,ITGB1
HGF Signaling	0.268	0.00758	#NUM!	ITGB1
p70S6K Signaling	0.268	0.00758	#NUM!	RPS6
Gα12/13 Signaling	0.267	0.00752	#NUM!	CDH2
STAT3 Pathway	0.262	0.00741	#NUM!	TGFB2
Adipogenesis pathway	0.262	0.00741	#NUM!	RBBP4
IL-12 Signaling and Production in Macrophages	0.26	0.00735	#NUM!	TGFB2
White Adipose Tissue Browning Pathway	0.26	0.00735	#NUM!	LDHA
Neuroinflammation Signaling Pathway	0.26	0.00635	#NUM!	HLA-A,TGFB2
MSP-RON Signaling In Cancer Cells Pathway	0.256	0.00725	#NUM!	ITGB1
Iron homeostasis signaling pathway	0.254	0.00719	#NUM!	PCBP1
Insulin Receptor Signaling	0.252	0.00714	#NUM!	CRK
Role of Macrophages, Fibroblasts and Endothelial Cells in Rheumatoid Arthritis	0.246	0.00615	#NUM!	2210010C04Rik,MIF
Dilated Cardiomyopathy Signaling Pathway	0.24	0.00685	#NUM!	MYH10
Cellular Effects of Sildenafil (Viagra)	0.234	0.00671	#NUM!	MYH10
Factors Promoting Cardiogenesis in Vertebrates	0.231	0.00662	#NUM!	TGFB2
Role of Pattern Recognition Receptors in Recognition of Bacteria and Viruses	0.223	0.00641	#NUM!	TGFB2
Necroptosis Signaling Pathway	0.22	0.00637	#NUM!	VDAC1
HOTAIR Regulatory Pathway	0.21	0.00613	#NUM!	RBBP4
Mitochondrial Dysfunction	0.2	0.00588	#NUM!	VDAC1
Glioblastoma Multiforme Signaling	0.199	0.00585	#NUM!	Cdc42
Glucocorticoid Receptor Signaling	0	0.00516	#NUM!	A2M,HLA-A,TGFB2
NRF2-mediated Oxidative Stress Response	0	0.00422	#NUM!	CCT7
Hepatic Cholestasis	0	0.00529	#NUM!	TGFB2
Erythropoietin Signaling Pathway	0	0.00565	#NUM!	TGFB2
CCR5 Signaling in Macrophages	0	0.00419	#NUM!	GNB2,GNB4
Calcium-induced T Lymphocyte Apoptosis	0	0.00228	#NUM!	HLA-A

© 2021. Kim H et al. Published online at http://www.aginganddisease.org/EN/10.14336/AD.2021.0621

SUPPLEMENTARY DATA

Cytotoxic T Lymphocyte-mediated Apoptosis of Target Cells	0	0.0024 8	#NUM !	HLA-A
IL-17 Signaling	0	0.0053 5	#NUM !	TGFB2
CTLA4 Signaling in Cytotoxic T Lymphocytes	0	0.0021 9	#NUM !	HLA-A
T Helper Cell Differentiation	0	0.0022 3	#NUM !	HLA-A
CD28 Signaling in T Helper Cells	0	0.0040 4	#NUM !	ARPC4,HLA-A
Dendritic Cell Maturation	0	0.0017 6	#NUM !	HLA-A
iCOS-iCOSL Signaling in T Helper Cells	0	0.0020 6	#NUM !	HLA-A
CREB Signaling in Neurons	0	0.0049 8	#NUM !	GNB2,GNB4,TGFB2
Type I Diabetes Mellitus Signaling	0	0.0020 5	#NUM !	HLA-A
Allograft Rejection Signaling	0	0.0021 7	#NUM !	HLA-A
Autoimmune Thyroid Disease Signaling	0	0.0023 2	#NUM !	HLA-A
Graft-versus-Host Disease Signaling	0	0.0023 6	#NUM !	HLA-A
Production of Nitric Oxide and Reactive Oxygen Species in Macrophages	0	0.0052 4	#NUM !	Cdc42
Communication between Innate and Adaptive Immune Cells	0	0.0021 1	#NUM !	HLA-A
Altered T Cell and B Cell Signaling in Rheumatoid Arthritis	0	0.0021 3	#NUM !	HLA-A
Regulation of IL-2 Expression in Activated and Anergic T Lymphocytes	0	0.0021 7	#NUM !	TGFB2
Nur77 Signaling in T Lymphocytes	0	0.0020 4	#NUM !	HLA-A
PKCθ Signaling in T Lymphocytes	0	0.0018 7	#NUM !	HLA-A
Antiproliferative Role of TOB in T Cell Signaling	0	0.0024 8	#NUM !	TGFB2
OX40 Signaling Pathway	0	0.0021 9	#NUM !	HLA-A
Granulocyte Adhesion and Diapedesis	0	0.0052 9	#NUM !	ITGB1
Xenobiotic Metabolism Signaling	0	0.0034 6	#NUM !	ALDH16A1
B Cell Receptor Signaling	0	0.0051 5	#NUM !	CFL2
NF-κB Signaling	0	0.0018 2	#NUM !	CSNK2A1
T Cell Receptor Signaling	0	0.0033 8	#NUM !	HLA-A,ITGB1
Autophagy	0	0.0046 9	#NUM !	TGFB2
T Cell Exhaustion Signaling Pathway	0	0.0018 4	#NUM !	HLA-A

© 2021. Kim H et al. Published online at http://www.aginganddisease.org/EN/10.14336/AD.2021.0621

Systemic Lupus Erythematosus In T Cell Signaling Pathway	0	0.0032 4	#NUM !	Cdc42,HLA-A
Systemic Lupus Erythematosus In B Cell Signaling Pathway	0	0.0036 1	#NUM !	TGFB2
Senescence Pathway	0	0.0033 7	#NUM !	TGFB2
Xenobiotic Metabolism CAR Signaling Pathway	0	0.0052 9	#NUM !	ALDH16A1
Xenobiotic Metabolism PXR Signaling Pathway	0	0.0052 1	#NUM !	ALDH16A1

Note: Applicable data are numbers of arteries with percentages in parentheses.

Supplementary Tables 3. IPA analyses on proteins completely unique to P15 EVs with P value < 0.05.

Ingenuity Canonical Pathways	-log (p-value)	Ratio	z-score	Molecules
Regulation of eIF4 and p70S6K Signaling	6.24	0.044 7	#NUM !	ITGA3,PABPC1,PPP2R2A,PPP2R2B,RPS10,RPS2,RPS24,RRAS
Signaling by Rho Family GTPases	4.94	0.029 9	#NUM !	CDH13,CDH8,GNAI2,IQGAP1,ITGA3,MYL9,ROCK1,ROCK2
14-3-3-mediated Signaling	4.93	0.047 2	#NUM !	PDCD6IP,RRAS,TUBB2A,TUBB2B,YWHAB,YWHAQ
p70S6K Signaling	4.84	0.045 5	#NUM !	GNAI2,PPP2R2A,PPP2R2B,RRAS,YWHAB,YWHAQ
Gα12/13 Signaling	4.82	0.045 1	#NUM !	CDH13,CDH8,MYL9,ROCK1,ROCK2,RRAS
Chemokine Signaling	4.77	0.062 5	#NUM !	CAMK2D,GNAI2,MPRIP,ROCK2,RRAS
Iron homeostasis signaling pathway	4.71	0.043 2	#NUM !	ATP6V1D,ATP6V1H,CUL1,HBB,Hbb-b1,Hbb-b2
Thrombin Signaling	4.68	0.033 3	#NUM !	CAMK2D,GNAI2,MPRIP,MYL9,ROCK1,ROCK2,RRAS
HIPPO signaling	4.65	0.058 8	#NUM !	CUL1,PPP2R2A,PPP2R2B,YWHAB,YWHAQ
ERK/MAPK Signaling	4.63	0.032 7	#NUM !	ITGA3,PPP2R2A,PPP2R2B,RRAS,STAT1,YWHAB,YWHAQ
RhoGDI Signaling	4.62	0.032 6	#NUM !	CDH13,CDH8,GNAI2,ITGA3,MYL9,ROCK1,ROCK2
Semaphorin Neuronal Repulsive Signaling Pathway	4.51	0.039 7	#NUM !	ITGA3,MPRIP,MYL9,ROCK1,ROCK2,RRAS
Axonal Guidance Signaling	4.48	0.019 8	#NUM !	ADAMTS7,EPHA5,GNAI2,ITGA3,MYL9,ROCK1,ROCK2,RRAS,TUBB2A,TUBB2B
Estrogen Receptor Signaling	4.48	0.022 3	#NUM !	ATP5F1B,CARM1,GNAI2,MMP19,MPRIP,MYL9,ROCK1,ROCK2,RRAS
Actin Cytoskeleton Signaling	4.26	0.028 6	#NUM !	IQGAP1,ITGA3,MPRIP,MYL9,ROCK1,ROCK2,RRAS
PI3K/AKT Signaling	3.84	0.030 2	#NUM !	ITGA3,PPP2R2A,PPP2R2B,RRAS,YWHAB,YWHAQ
Ephrin Receptor Signaling	3.82	0.029 9	#NUM !	EPHA5,GNAI2,ITGA3,ROCK1,ROCK2,RRAS

© 2021. Kim H et al. Published online at http://www.aginganddisease.org/EN/10.14336/AD.2021.0621

SUPPLEMENTARY DATA

IL-8 Signaling	3.7	0.0284	#NUM!	GNAI2,IQGAP1,MYL9,ROCK1,ROCK2,RRAS
Protein Kinase A Signaling	3.7	0.0199	#NUM!	CAMK2D,GNAI2,MYL9,PTPRK,ROCK1,ROCK2,YWHAB,YWHAQ
mTOR Signaling	3.69	0.0283	#NUM!	PPP2R2A,PPP2R2B,RPS10,RPS2,RPS24,RRAS
CCR3 Signaling in Eosinophils	3.69	0.037	#NUM!	GNAI2,MPRIP,ROCK1,ROCK2,RRAS
Integrin Signaling	3.68	0.0282	#NUM!	CAPN2,ITGA3,MPRIP,MYL9,ROCK1,RRAS
EIF2 Signaling	3.56	0.0268	#NUM!	PABPC1,RPS10,RPS2,RPS24,RRAS,WARS1
Epithelial Adherens Junction Signaling	3.37	0.0316	#NUM!	IQGAP1,MYL9,RRAS,TUBB2A,TUBB2B
Actin Nucleation by ARP-WASP Complex	3.28	0.043	#NUM!	ITGA3,ROCK1,ROCK2,RRAS
CXCR4 Signaling	3.26	0.0299	#NUM!	GNAI2,MYL9,ROCK1,ROCK2,RRAS
Germ Cell-Sertoli Cell Junction Signaling	3.22	0.0292	#NUM!	IQGAP1,ITGA3,RRAS,TUBB2A,TUBB2B
Apoptosis Signaling	3.1	0.0385	#NUM!	CAPN2,LMNA,ROCK1,RRAS
Ephrin A Signaling	3.06	0.0638	#NUM!	EPHA5,ROCK1,ROCK2
Leukocyte Extravasation Signaling	2.98	0.0259	#NUM!	GNAI2,ITGA3,MMP19,ROCK1,ROCK2
CDK5 Signaling	2.98	0.0357	#NUM!	ITGA3,PPP2R2A,PPP2R2B,RRAS
Cell Cycle: G2/M DNA Damage Checkpoint Regulation	2.98	0.06	#NUM!	CUL1,YWHAB,YWHAQ
Synaptic Long Term Depression	2.97	0.0258	#NUM!	GNAI2,NPR3,PPP2R2A,PPP2R2B,RRAS
Gap Junction Signaling	2.93	0.0253	#NUM!	GNAI2,NPR3,RRAS,TUBB2A,TUBB2B
Regulation of Actin-based Motility by Rho	2.92	0.0345	#NUM!	ITGA3,MPRIP,MYL9,ROCK1
RhoA Signaling	2.82	0.0323	#NUM!	MPRIP,MYL9,ROCK1,ROCK2
Synaptogenesis Signaling Pathway	2.81	0.0192	#NUM!	CAMK2D,CDH13,CDH8,EPHA5,PAFAH1B1,RRAS
Remodeling of Epithelial Adherens Junctions	2.6	0.0441	#NUM!	IQGAP1,TUBB2A,TUBB2B
GM-CSF Signaling	2.56	0.0429	#NUM!	CAMK2D,RRAS,STAT1
ERK5 Signaling	2.53	0.0417	#NUM!	RRAS,YWHAB,YWHAQ
Ephrin B Signaling	2.53	0.0417	#NUM!	GNAI2,ROCK1,ROCK2
Necroptosis Signaling Pathway	2.45	0.0255	#NUM!	CAMK2D,CAPN2,STAT1,UBC
Cardiac Hypertrophy Signaling	2.43	0.0194	#NUM!	GNAI2,MYL9,ROCK1,ROCK2,RRAS
Phagosome Maturation	2.43	0.0252	#NUM!	ATP6V1D,ATP6V1H,TUBB2A,TUBB2B
Inhibition of ARE-Mediated mRNA Degradation Pathway	2.41	0.0248	#NUM!	PPP2R2A,PPP2R2B,YWHAB,YWHAQ
Asparagine Biosynthesis I	2.4	1	#NUM!	ASNS
Renal Cell Carcinoma Signaling	2.4	0.0375	#NUM!	RRAS,Ubb,UBC

© 2021. Kim H et al. Published online at http://www.aginganddisease.org/EN/10.14336/AD.2021.0621

Cyclins and Cell Cycle Regulation	2.34	0.0357	#NUM!	CUL1,PPP2R2A,PPP2R2B
PEDF Signaling	2.34	0.0357	#NUM!	ROCK1,ROCK2,RRAS
Wnt/β-catenin Signaling	2.3	0.0231	#NUM!	PPP2R2A,PPP2R2B,Ubb,UBC
Regulation of Cellular Mechanics by Calpain Protease	2.27	0.0337	#NUM!	CAPN2,ITGA3,RRAS
Erythropoietin Signaling Pathway	2.26	0.0226	#NUM!	HBB,Hbb-b1,Hbb-b2,RRAS
Ceramide Signaling	2.26	0.0333	#NUM!	PPP2R2A,PPP2R2B,RRAS
Xenobiotic Metabolism Signaling	2.23	0.0173	#NUM!	CAMK2D,ESD,PPP2R2A,PPP2R2B,RRAS
Hepatic Fibrosis Signaling Pathway	2.19	0.0143	#NUM!	GNAI2,ITGA3,MYL9,ROCK1,ROCK2,RRAS
VEGF Signaling	2.14	0.0303	#NUM!	ROCK1,ROCK2,RRAS
Formaldehyde Oxidation II (Glutathione-dependent)	2.1	0.5	#NUM!	ESD
IGF-1 Signaling	2.08	0.0288	#NUM!	RRAS,YWHAB,YWHAQ
Coronavirus Pathogenesis Pathway	2.06	0.0197	#NUM!	RPS10,RPS2,RPS24,STAT1
Telomerase Signaling	2.05	0.028	#NUM!	PPP2R2A,PPP2R2B,RRAS
Sertoli Cell-Sertoli Cell Junction Signaling	2.03	0.0194	#NUM!	ITGA3,RRAS,TUBB2A,TUBB2B
Cell Cycle Regulation by BTG Family Proteins	2.02	0.0541	#NUM!	PPP2R2A,PPP2R2B
Role of MAPK Signaling in Promoting the Pathogenesis of Influenza	2	0.0268	#NUM!	ATP6V1D,ATP6V1H,RRAS
Agranulocyte Adhesion and Diapedesis	1.98	0.0187	#NUM!	GNAI2,ITGA3,MMP19,MYL9
FAK Signaling	1.94	0.0256	#NUM!	CAPN2,ITGA3,RRAS
PAK Signaling	1.94	0.0254	#NUM!	ITGA3,MYL9,RRAS
NGF Signaling	1.94	0.0254	#NUM!	ROCK1,ROCK2,RRAS
Cholecystokinin/Gastrin-mediated Signaling	1.92	0.0252	#NUM!	ROCK1,ROCK2,RRAS
Oncostatin M Signaling	1.9	0.0465	#NUM!	RRAS,STAT1
Coronavirus Replication Pathway	1.86	0.0444	#NUM!	TUBB2A,TUBB2B
Reelin Signaling in Neurons	1.86	0.0238	#NUM!	CAMK2D,ITGA3,PAFAH1B1
GABA Receptor Signaling	1.81	0.0229	#NUM!	GNAI2,Ubb,UBC
AMPK Signaling	1.8	0.0165	#NUM!	GNAI2,PPP2R2A,PPP2R2B,RAB2A
Rac Signaling	1.75	0.0217	#NUM!	IQGAP1,ITGA3,RRAS
Gαi Signaling	1.75	0.0217	#NUM!	GNAI2,NPR3,RRAS
MSP-RON Signaling In Cancer Cells Pathway	1.75	0.0217	#NUM!	RRAS,YWHAB,YWHAQ
Hereditary Breast Cancer Signaling	1.72	0.0211	#NUM!	RRAS,Ubb,UBC

© 2021. Kim H et al. Published online at http://www.aginganddisease.org/EN/10.14336/AD.2021.0621

Endocannabinoid Cancer Inhibition Pathway	1.71	0.021	#NUM!	GNAI2,ROCK1,ROCK2
Creatine-phosphate Biosynthesis	1.71	0.2	#NUM!	CKB
Cardiac Hypertrophy Signaling (Enhanced)	1.69	0.0112	#NUM!	CAMK2D,GNAI2,ITGA3,ROCK1,ROCK2,RRAS
Dilated Cardiomyopathy Signaling Pathway	1.69	0.0205	#NUM!	CAMK2D,LMNA,MYL9
Cellular Effects of Sildenafil (Viagra)	1.67	0.0201	#NUM!	MPRIP,MYL9,PABPC4
CNTF Signaling	1.67	0.0351	#NUM!	RRAS,STAT1
Role of CHK Proteins in Cell Cycle Checkpoint Control	1.67	0.0351	#NUM!	PPP2R2A,PPP2R2B
Factors Promoting Cardiogenesis in Vertebrates	1.65	0.0199	#NUM!	CAMK2D,ROCK1,ROCK2
Colorectal Cancer Metastasis Signaling	1.65	0.0149	#NUM!	GNAI2,MMP19,RRAS,STAT1
PCP pathway	1.62	0.0333	#NUM!	ROCK1,ROCK2
Protein Ubiquitination Pathway	1.62	0.0145	#NUM!	CUL1,HSPD1,Ubb,UBC
G-Protein Coupled Receptor Signaling	1.61	0.0145	#NUM!	CAMK2D,GNAI2,NPR3,RRAS
Semaphorin Signaling in Neurons	1.6	0.0323	#NUM!	ROCK1,ROCK2
Huntington's Disease Signaling	1.59	0.0142	#NUM!	ATP5F1B,CAPN2,Ubb,UBC
Thrombopoietin Signaling	1.59	0.0317	#NUM!	RRAS,STAT1
HOTAIR Regulatory Pathway	1.57	0.0184	#NUM!	MMP19,ROCK1,ROCK2
Glucocorticoid Receptor Signaling	1.55	0.0103	#NUM!	ATP5F1B,KRT6B,KRT73,KRT75,RRAS,STAT1
Mitotic Roles of Polo-Like Kinase	1.55	0.0303	#NUM!	PPP2R2A,PPP2R2B
Gαq Signaling	1.52	0.0176	#NUM!	GNAI2,ROCK1,ROCK2
Breast Cancer Regulation by Stathmin1	1.51	0.0101	#NUM!	CAMK2D,PPP2R2A,PPP2R2B,RRAS,TUBB2A,TUBB2B
Senescence Pathway	1.51	0.0135	#NUM!	CAPN2,PPP2R2A,PPP2R2B,RRAS
Role of JAK1 and JAK3 in γc Cytokine Signaling	1.51	0.029	#NUM!	RRAS,STAT1
Cardiac β-adrenergic Signaling	1.49	0.0172	#NUM!	GNAI2,PPP2R2A,PPP2R2B
Melatonin Signaling	1.48	0.0278	#NUM!	CAMK2D,GNAI2
Tight Junction Signaling	1.47	0.0169	#NUM!	MYL9,PPP2R2A,PPP2R2B
Dopamine-DARPP32 Feedback in cAMP Signaling	1.45	0.0166	#NUM!	GNAI2,PPP2R2A,PPP2R2B
Systemic Lupus Erythematosus In T Cell Signaling Pathway	1.44	0.00972	#NUM!	GNAI2,PPP2R2A,PPP2R2B,ROCK1,ROCK2,RRAS
Antiproliferative Role of Somatostatin Receptor 2	1.42	0.026	#NUM!	NPR3,RRAS
Dopamine Receptor Signaling	1.42	0.026	#NUM!	PPP2R2A,PPP2R2B
NF-κB Activation by Viruses	1.41	0.0256	#NUM!	ITGA3,RRAS

SUPPLEMENTARY DATA

Toll-like Receptor Signaling	1.41	0.025 6	#NUM !	Ubb,UBC
GNRH Signaling	1.41	0.016	#NUM !	CAMK2D,GNAI2,RRAS
IL-3 Signaling	1.4	0.025 3	#NUM !	RRAS,STAT1
FLT3 Signaling in Hematopoietic Progenitor Cells	1.39	0.025	#NUM !	RRAS,STAT1
Production of Nitric Oxide and Reactive Oxygen Species in Macrophages	1.39	0.015 7	#NUM !	PPP2R2A,PPP2R2B,STAT1
Role of Macrophages, Fibroblasts and Endothelial Cells in Rheumatoid Arthritis	1.39	0.012 3	#NUM !	CAMK2D,ROCK1,ROCK2,RRAS
BEX2 Signaling Pathway	1.38	0.024 7	#NUM !	PPP2R2A,PPP2R2B
JAK/Stat Signaling	1.37	0.024 4	#NUM !	RRAS,STAT1
ILK Signaling	1.35	0.015 2	#NUM !	MYL9,PPP2R2A,PPP2R2B
Prolactin Signaling	1.34	0.023 3	#NUM !	RRAS,STAT1
PDGF Signaling	1.34	0.023 3	#NUM !	RRAS,STAT1
HIF1α Signaling	1.3	0.014 4	#NUM !	CAMK2D,MMP19,RRAS
Calcium Signaling	1.26	0.013 9	#NUM !	CAMK2D,MYL9,TPM3
Death Receptor Signaling	1.25	0.020 8	#NUM !	LMNA,ROCK1
ATM Signaling	1.24	0.020 6	#NUM !	PPP2R2A,PPP2R2B
Role of NFAT in Cardiac Hypertrophy	1.24	0.013 6	#NUM !	CAMK2D,GNAI2,RRAS
UVA-Induced MAPK Signaling	1.24	0.020 4	#NUM !	RRAS,STAT1
Apelin Cardiomyocyte Signaling Pathway	1.23	0.020 2	#NUM !	GNAI2,MYL9
cAMP-mediated signaling	1.2	0.013 1	#NUM !	CAMK2D,GNAI2,NPR3
Dermatan Sulfate Degradation (Metazoa)	1.18	0.058 8	#NUM !	IDS
α-Adrenergic Signaling	1.18	0.018 9	#NUM !	GNAI2,RRAS
Paxillin Signaling	1.16	0.018 5	#NUM !	ITGA3,RRAS
Bladder Cancer Signaling	1.11	0.017 2	#NUM !	MMP19,RRAS
Role of Tissue Factor in Cancer	1.11	0.017 2	#NUM !	ITGA3,RRAS
Neuregulin Signaling	1.1	0.017 1	#NUM !	ITGA3,RRAS
MSP-RON Signaling In Macrophages Pathway	1.1	0.017 1	#NUM !	RRAS,STAT1
Renin-Angiotensin Signaling	1.08	0.016 7	#NUM !	RRAS,STAT1
IL-15 Production	1.07	0.016 3	#NUM !	EPHA5,STAT1
Endocannabinoid Developing Neuron Pathway	1.07	0.016 3	#NUM !	GNAI2,RRAS

© 2021. Kim H et al. Published online at http://www.aginganddisease.org/EN/10.14336/AD.2021.0621

Glioma Signaling	1.06	0.016 1	#NUM !	CAMK2D,RRAS
Insulin Secretion Signaling Pathway	1.04	0.011 2	#NUM !	CAMK2D,PABPC1,STAT1
IL-22 Signaling	1.04	0.041 7	#NUM !	STAT1
Synaptic Long Term Potentiation	1.03	0.015 5	#NUM !	CAMK2D,RRAS
G Beta Gamma Signaling	1.03	0.015 5	#NUM !	GNAI2,RRAS
P2Y Purigenic Receptor Signaling Pathway	1.03	0.015 5	#NUM !	GNAI2,RRAS
Role of JAK family kinases in IL-6-type Cytokine Signaling	1.02	0.04	#NUM !	STAT1
fMLP Signaling in Neutrophils	1.02	0.015 3	#NUM !	GNAI2,RRAS
HGF Signaling	1.01	0.015 2	#NUM !	ITGA3,RRAS
Opioid Signaling Pathway	1.01	0.010 9	#NUM !	CAMK2D,GNAI2,RRAS
Role of JAK1, JAK2 and TYK2 in Interferon Signaling	1.01	0.038 5	#NUM !	STAT1
Molecular Mechanisms of Cancer	0.996	0.008 99	#NUM !	CAMK2D,GNAI2,ITGA3,RRAS
Apelin Endothelial Signaling Pathway	0.975	0.014 4	#NUM !	GNAI2,RRAS
PI3K Signaling in B Lymphocytes	0.955	0.014	#NUM !	CAMK2D,RRAS
Corticotropin Releasing Hormone Signaling	0.928	0.013 4	#NUM !	GNAI2,NPR3
PTEN Signaling	0.921	0.013 3	#NUM !	ITGA3,RRAS
Relaxin Signaling	0.907	0.013 1	#NUM !	GNAI2,NPR3
Role of JAK2 in Hormone-like Cytokine Signaling	0.896	0.029 4	#NUM !	STAT1
Pyrimidine Ribonucleotides Interconversion	0.896	0.029 4	#NUM !	CANT1
IL-9 Signaling	0.886	0.028 6	#NUM !	STAT1
Pyrimidine Ribonucleotides De Novo Biosynthesis	0.873	0.027 8	#NUM !	CANT1
Interferon Signaling	0.873	0.027 8	#NUM !	STAT1
tRNA Charging	0.842	0.025 6	#NUM !	WARS1
Inhibition of Matrix Metalloproteases	0.842	0.025 6	#NUM !	MMP19
Mechanisms of Viral Exit from Host Cells	0.821	0.024 4	#NUM !	PDCD6IP
D-myo-inositol (1,4,5,6)-Tetrakisphosphate Biosynthesis	0.807	0.011 3	#NUM !	PPP2R2A,PPP2R2B
D-myo-inositol (3,4,5,6)-tetrakisphosphate Biosynthesis	0.807	0.011 3	#NUM !	PPP2R2A,PPP2R2B
Tumor Microenvironment Pathway	0.799	0.011 2	#NUM !	MMP19,RRAS
G Protein Signaling Mediated by Tubby	0.793	0.022 7	#NUM !	GNAI2
Retinol Biosynthesis	0.793	0.022 7	#NUM !	ESD

© 2021. Kim H et al. Published online at http://www.aginganddisease.org/EN/10.14336/AD.2021.0621

iNOS Signaling	0.767	0.0213	#NUM!	STAT1
nNOS Signaling in Neurons	0.767	0.0213	#NUM!	CAPN2
T Cell Exhaustion Signaling Pathway	0.767	0.00735	#NUM!	PPP2R2A,PPP2R2B,RRAS,STAT1
3-phosphoinositide Degradation	0.762	0.0106	#NUM!	PPP2R2A,PPP2R2B
Granulocyte Adhesion and Diapedesis	0.762	0.0106	#NUM!	GNAI2,MMP19
Xenobiotic Metabolism CAR Signaling Pathway	0.762	0.0106	#NUM!	PPP2R2A,PPP2R2B
Apelin Muscle Signaling Pathway	0.759	0.0208	#NUM!	GNAI2
Endothelin-1 Signaling	0.754	0.0105	#NUM!	GNAI2,RRAS
Spliceosomal Cycle	0.752	0.0204	#NUM!	SF3B6
Xenobiotic Metabolism PXR Signaling Pathway	0.752	0.0104	#NUM!	CAMK2D,ESD
Cdc42 Signaling	0.75	0.00723	#NUM!	IQGAP1,ITGA3,MPRIP,MYL9
Clathrin-mediated Endocytosis Signaling	0.747	0.0104	#NUM!	Ubb,UBC
D-myo-inositol-5-phosphate Metabolism	0.747	0.0104	#NUM!	PPP2R2A,PPP2R2B
Hepatic Fibrosis / Hepatic Stellate Cell Activation	0.745	0.0103	#NUM!	MYL9,STAT1
B Cell Receptor Signaling	0.745	0.0103	#NUM!	CAMK2D,RRAS
Melanoma Signaling	0.742	0.02	#NUM!	RRAS
UVC-Induced MAPK Signaling	0.735	0.0196	#NUM!	RRAS
Amyloid Processing	0.735	0.0196	#NUM!	CAPN2
Natural Killer Cell Signaling	0.728	0.0101	#NUM!	ROCK1,RRAS
Adrenomedullin signaling pathway	0.728	0.0101	#NUM!	NPR3,RRAS
3-phosphoinositide Biosynthesis	0.714	0.00985	#NUM!	PPP2R2A,PPP2R2B
EGF Signaling	0.706	0.0182	#NUM!	STAT1
Cancer Drug Resistance By Drug Efflux	0.686	0.0172	#NUM!	RRAS
Autophagy	0.684	0.00939	#NUM!	PPP2R2A,PPP2R2B
Endometrial Cancer Signaling	0.672	0.0167	#NUM!	RRAS
SPINK1 Pancreatic Cancer Pathway	0.672	0.0167	#NUM!	CPA4
IL-2 Signaling	0.666	0.0164	#NUM!	RRAS
Antiproliferative Role of TOB in T Cell Signaling	0.664	0.00743	#NUM!	CUL1,PABPC1,PABPC4
Activation of IRF by Cytosolic Pattern Recognition Receptors	0.642	0.0154	#NUM!	STAT1
ErbB2-ErbB3 Signaling	0.642	0.0154	#NUM!	RRAS

© 2021. Kim H et al. Published online at http://www.aginganddisease.org/EN/10.14336/AD.2021.0621

Role of PI3K/AKT Signaling in the Pathogenesis of Influenza	0.636	0.0152	#NUM!	GNAI2
ErbB4 Signaling	0.625	0.0147	#NUM!	RRAS
Superpathway of Inositol Phosphate Compounds	0.625	0.00858	#NUM!	PPP2R2A,PPP2R2B
Cell Cycle: G1/S Checkpoint Regulation	0.625	0.0147	#NUM!	CUL1
SPINK1 General Cancer Pathway	0.62	0.0145	#NUM!	RRAS
Agrin Interactions at Neuromuscular Junction	0.614	0.0143	#NUM!	RRAS
Growth Hormone Signaling	0.609	0.0141	#NUM!	STAT1
Glioma Invasiveness Signaling	0.599	0.0137	#NUM!	RRAS
GPCR-Mediated Integration of Enteroendocrine Signaling Exemplified by an L Cell	0.599	0.0137	#NUM!	GNAI2
Caveolar-mediated Endocytosis Signaling	0.588	0.0133	#NUM!	ITGA3
Phospholipase C Signaling	0.585	0.00613	#NUM!	ITGA3,MPRIP,MYL9,RRAS
GDNF Family Ligand-Receptor Interactions	0.583	0.0132	#NUM!	RRAS
Neurotrophin/TRK Signaling	0.583	0.0132	#NUM!	RRAS
Macropinocytosis Signaling	0.583	0.0132	#NUM!	RRAS
IL-15 Signaling	0.578	0.013	#NUM!	RRAS
Angiopoietin Signaling	0.578	0.013	#NUM!	RRAS
Sperm Motility	0.573	0.00787	#NUM!	EPHA5,GNAI2
IL-7 Signaling Pathway	0.573	0.0128	#NUM!	STAT1
Thyroid Cancer Signaling	0.569	0.0127	#NUM!	RRAS
Estrogen-Dependent Breast Cancer Signaling	0.569	0.0127	#NUM!	RRAS
Role of BRCA1 in DNA Damage Response	0.564	0.0125	#NUM!	STAT1
Role of MAPK Signaling in the Pathogenesis of Influenza	0.559	0.0123	#NUM!	RRAS
VEGF Family Ligand-Receptor Interactions	0.545	0.0119	#NUM!	RRAS
FcγRIIB Signaling in B Lymphocytes	0.542	0.0118	#NUM!	RRAS
LPS-stimulated MAPK Signaling	0.542	0.0118	#NUM!	RRAS
Role of Hypercytokinemia/hyperchemokinemia in the Pathogenesis of Influenza	0.538	0.0116	#NUM!	STAT1
Apelin Adipocyte Signaling Pathway	0.538	0.0116	#NUM!	GNAI2
BMP signaling pathway	0.533	0.0115	#NUM!	RRAS
Systemic Lupus Erythematosus In B Cell Signaling Pathway	0.521	0.00722	#NUM!	RRAS,STAT1

© 2021. Kim H et al. Published online at http://www.aginganddisease.org/EN/10.14336/AD.2021.0621

Acute Myeloid Leukemia Signaling	0.517	0.011	#NUM!	RRAS
Crosstalk between Dendritic Cells and Natural Killer Cells	0.517	0.011	#NUM!	CAMK2D
IL-4 Signaling	0.513	0.0109	#NUM!	RRAS
Melanocyte Development and Pigmentation Signaling	0.504	0.0106	#NUM!	RRAS
Non-Small Cell Lung Cancer Signaling	0.504	0.0106	#NUM!	RRAS
IL-1 Signaling	0.504	0.0106	#NUM!	GNAI2
ErbB Signaling	0.504	0.0106	#NUM!	RRAS
TGF-β Signaling	0.498	0.0104	#NUM!	RRAS
Neuropathic Pain Signaling In Dorsal Horn Neurons	0.479	0.0099	#NUM!	CAMK2D
NER (Nucleotide Excision Repair, Enhanced Pathway)	0.472	0.00971	#NUM!	DDB1
Virus Entry via Endocytic Pathways	0.47	0.00962	#NUM!	RRAS
Mouse Embryonic Stem Cell Pluripotency	0.47	0.00962	#NUM!	RRAS
Chronic Myeloid Leukemia Signaling	0.46	0.00935	#NUM!	RRAS
PPAR Signaling	0.46	0.00935	#NUM!	RRAS
Oxidative Phosphorylation	0.452	0.00917	#NUM!	ATP5F1B
Prostate Cancer Signaling	0.444	0.00893	#NUM!	RRAS
Amyotrophic Lateral Sclerosis Signaling	0.434	0.0087	#NUM!	CAPN2
GPCR-Mediated Nutrient Sensing in Enteroendocrine Cells	0.432	0.00862	#NUM!	GNAI2
Fc Epsilon RI Signaling	0.428	0.00855	#NUM!	RRAS
Sphingosine-1-phosphate Signaling	0.426	0.00847	#NUM!	GNAI2
Neuroprotective Role of THOP1 in Alzheimer's Disease	0.426	0.00847	#NUM!	HTRA3
p38 MAPK Signaling	0.426	0.00847	#NUM!	STAT1
Role of NANOG in Mammalian Embryonic Stem Cell Pluripotency	0.42	0.00833	#NUM!	RRAS
Tec Kinase Signaling	0.42	0.0054	#NUM!	GNAI2,ITGA3,STAT1
Gαs Signaling	0.417	0.00826	#NUM!	GNAI2
Th1 Pathway	0.415	0.0082	#NUM!	STAT1
Pancreatic Adenocarcinoma Signaling	0.403	0.00794	#NUM!	STAT1
Ferroptosis Signaling Pathway	0.403	0.00794	#NUM!	RRAS
IL-6 Signaling	0.398	0.00781	#NUM!	RRAS

© 2021. Kim H et al. Published online at http://www.aginganddisease.org/EN/10.14336/AD.2021.0621

SUPPLEMENTARY DATA

STAT3 Pathway	0.38	0.007 41	#NUM !	RRAS
IL-12 Signaling and Production in Macrophages	0.378	0.007 35	#NUM !	STAT1
Role of PKR in Interferon Induction and Antiviral Response	0.378	0.007 35	#NUM !	STAT1
Insulin Receptor Signaling	0.369	0.007 14	#NUM !	RRAS
CREB Signaling in Neurons	0.368	0.004 98	#NUM !	CAMK2D,GNAI2,RRAS
Xenobiotic Metabolism General Signaling Pathway	0.362	0.006 99	#NUM !	RRAS
Endocannabinoid Neuronal Synapse Pathway	0.353	0.006 8	#NUM !	GNAI2
Phagosome Formation	0.349	0.006 71	#NUM !	ITGA3
NAD Signaling Pathway	0.344	0.006 62	#NUM !	HSPD1
Ovarian Cancer Signaling	0.33	0.006 33	#NUM !	RRAS
Aldosterone Signaling in Epithelial Cells	0.319	0.006 1	#NUM !	HSPD1
Human Embryonic Stem Cell Pluripotency	0.314	0.006 02	#NUM !	GNAI2
HMGB1 Signaling	0.312	0.005 99	#NUM !	RRAS
Androgen Signaling	0.309	0.005 92	#NUM !	GNAI2
Mitochondrial Dysfunction	0.307	0.005 88	#NUM !	ATP5F1B
Glioblastoma Multiforme Signaling	0.305	0.005 85	#NUM !	RRAS
Th1 and Th2 Activation Pathway	0.304	0.005 81	#NUM !	STAT1
Acute Phase Response Signaling	0.282	0.005 41	#NUM !	RRAS
IL-17 Signaling	0.279	0.005 35	#NUM !	RRAS
Regulation Of The Epithelial Mesenchymal Transition By Growth Factors Pathway	0.271	0.005 21	#NUM !	RRAS
PPARα/RXRα Activation	0.269	0.005 18	#NUM !	RRAS
Regulation of the Epithelial-Mesenchymal Transition Pathway	0.267	0.005 13	#NUM !	RRAS
RAR Activation	0.265	0.005 1	#NUM !	CARM1
CTLA4 Signaling in Cytotoxic T Lymphocytes	0.265	0.004 39	#NUM !	PPP2R2A,PPP2R2B
HER-2 Signaling in Breast Cancer	0.265	0.005 1	#NUM !	RRAS
Regulation of IL-2 Expression in Activated and Anergic T Lymphocytes	0.26	0.004 34	#NUM !	PABPC4,RRAS
Type I Diabetes Mellitus Signaling	0.237	0.004 11	#NUM !	HSPD1,STAT1
Osteoarthritis Pathway	0.216	0.004 27	#NUM !	ITGA3

© 2021. Kim H et al. Published online at http://www.aginganddisease.org/EN/10.14336/AD.2021.0621

NRF2-mediated Oxidative Stress Response	0.213	0.00422	#NUM!	RRAS
Role of NFAT in Regulation of the Immune Response	0	0.00353	#NUM!	GNAI2,RRAS
CCR5 Signaling in Macrophages	0	0.0021	#NUM!	GNAI2
Calcium-induced T Lymphocyte Apoptosis	0	0.00228	#NUM!	CAPN2
T Helper Cell Differentiation	0	0.00223	#NUM!	STAT1
Dendritic Cell Maturation	0	0.00176	#NUM!	STAT1
iCOS-iCOSL Signaling in T Helper Cells	0	0.00206	#NUM!	CAMK2D
Systemic Lupus Erythematosus Signaling	0	0.00334	#NUM!	LSM2,RRAS
PKCθ Signaling in T Lymphocytes	0	0.00373	#NUM!	CAMK2D,RRAS
SAPK/JNK Signaling	0	0.00208	#NUM!	RRAS
NF-κB Signaling	0	0.00182	#NUM!	RRAS
T Cell Receptor Signaling	0	0.00338	#NUM!	ITGA3,RRAS
Sirtuin Signaling Pathway	0	0.00342	#NUM!	ATP5F1B
Neuroinflammation Signaling Pathway	0	0.00317	#NUM!	STAT1

Supplementary Table 4. A list of miRNAs found in P5 and P15 EVs.

**miR_name**	**P5 EVs (Normalized)**	**P15 EVs (Normalized)**	**log2 (fold_change)**	**-LOG10 (p value)**
hsa-miR-21-5p	12,738	4,062	-1.65	256.0000
hsa-miR-143-3p_R+1	3,256	3,084	-0.08	211.8913
hsa-miR-27b-3p	2,664	2,551	-0.06	168.2356
hsa-miR-125b-5p	2,129	10,210	2.26	256.0000
hsa-miR-221-3p	1,951	7,283	1.90	142.5064
hsa-miR-92a-3p	1,667	4,026	1.27	8.1280
hsa-miR-100-5p	1,637	1,740	0.09	80.2324
hsa-miR-22-3p	1,232	1,758	0.51	21.5989
hsa-miR-222-3p_R+2	1,160	1,648	0.51	20.7474
hsa-miR-27a-3p	1,080	993	-0.12	73.3398
hsa-miR-10b-5p_R-1	911	91	-3.32	256.0000
hsa-miR-574-3p	886	10,929	3.63	256.0000
hsa-miR-23a-3p_R+1	884	433	-1.03	256.0000
hsa-miR-19b-3p	834	22	-5.24	256.0000
hsa-miR-145-5p	822	3,082	1.91	59.7674
hsa-miR-29a-3p_R-1	727	1,167	0.68	6.5861
hsa-miR-199a-5p	648	652	0.01	36.4892
hsa-let-7i-5p	638	705	0.14	28.4481
hsa-miR-26a-5p	618	524	-0.24	49.1328
hsa-miR-19a-3p	561	8	-6.08	256.4894
hsa-miR-125a-5p_R-1	535	1,365	1.35	4.8748
hsa-miR-99a-5p	494	298	-0.73	64.5195
hsa-let-7a-5p	476	373	-0.35	43.3865
hsa-let-7f-5p	460	139	-1.73	107.5839
hsa-miR-199a-3p_R-1	446	547	0.29	14.7124
hsa-miR-24-3p_R+1	430	233	-0.89	63.1291

© 2021. Kim H et al. Published online at http://www.aginganddisease.org/EN/10.14336/AD.2021.0621

hsa-miR-148a-3p	406	39	-3.39	147.5019
hsa-miR-17-5p	400	39	-3.36	144.8215
hsa-miR-106a-5p_1ss1AC	400	39	-3.36	144.8215
hsa-let-7g-5p	389	652	0.74	2.7357
hsa-miR-30e-5p_R+2	385	204	-0.92	58.0917
hsa-miR-335-5p	368	313	-0.23	29.4145
hsa-miR-20a-5p	359	44	-3.04	122.5801
hsa-miR-99b-5p	355	1,237	1.80	19.6275
hsa-miR-30d-5p_R+2	342	575	0.75	2.4183
hsa-miR-146a-5p	308	12	-4.66	130.1735
PC-3p-9853_49	300	0	-inf	145.5249
hsa-miR-127-3p	291	4,728	4.02	256.0000
hsa-miR-320a-3p	270	2,190	3.02	142.0282
hsa-miR-26b-5p_R+1	265	98	-1.43	54.5313
hsa-miR-181a-5p	255	1,266	2.31	44.7892
hsa-miR-23b-3p_R+1	242	127	-0.93	37.2058
hsa-miR-16-5p	227	103	-1.13	39.8700
hsa-miR-30a-5p_R+2	227	397	0.81	1.2215
hsa-miR-191-5p	222	757	1.77	11.4670
hsa-miR-363-3p_R-1	214	0	-inf	103.7567
hsa-miR-424-5p_R-1	205	16	-3.71	78.1767
hsa-let-7b-5p	205	1,032	2.33	37.4713
hsa-let-7e-5p	201	85	-1.24	37.5436
hsa-miR-103a-3p	198	52	-1.92	50.5732
hsa-miR-1246_R+1	197	21	-3.23	70.1281
hsa-miR-31-5p_R+2	188	151	-0.32	16.9645
hsa-miR-20b-5p	180	0	-inf	87.2550
oga-miR-100_R+1	175	83	-1.07	29.7067
hsa-miR-423-3p	171	4,312	4.65	256.0000
hsa-miR-199b-5p	163	118	-0.46	17.3397
hsa-miR-484	163	827	2.35	30.4865
hsa-miR-30c-5p_R+1	154	290	0.91	0.4089
hsa-let-7d-5p	141	62	-1.18	25.8510
hsa-miR-142-5p_L+2R-1	137	47	-1.54	30.1646
hsa-miR-214-3p	128	275	1.10	0.1487
hsa-miR-152-3p	120	92	-0.39	11.9165
hsa-miR-93-5p	120	377	1.65	4.5259
hsa-miR-1-3p	118	9	-3.65	45.4655
hsa-miR-181b-5p_R+1	109	283	1.38	1.4477
hsa-miR-335-3p	107	84	-0.35	10.2013
hsa-miR-143-5p_R-1	94	67	-0.49	10.4808
hsa-miR-186-5p_R+1	94	149	0.66	1.2568
hsa-miR-302a-5p_L-3R+2	94	0	-inf	45.5446
bta-miR-1246_L-1R+2	90	43	-1.07	15.5055
cgr-miR-1260	90	52	-0.78	12.9518
hsa-miR-30b-5p	90	92	0.03	5.4313
hsa-miR-25-3p	90	220	1.29	0.7850
hsa-miR-34a-5p	86	8	-3.37	31.9783
hsa-miR-151b_R+3	81	84	0.05	4.7160
hsa-miR-365b-3p	79	59	-0.44	8.3064
hsa-miR-196a-5p	79	0	-inf	38.2736
oan-miR-16c-5p	77	4	-4.14	31.7610
hsa-miR-101-3p_R+1	77	20	-1.94	20.2587
hsa-miR-374a-5p	77	24	-1.71	18.3513
hsa-miR-15b-5p	77	48	-0.67	10.3311
efu-miR-9226_2ss4AG22GA	77	66	-0.23	6.6099
hsa-miR-193a-5p	73	717	3.30	54.5106
oan-miR-222b-3p_R+2	68	9	-2.90	23.2576
hsa-miR-130a-3p	68	28	-1.27	13.5375

© 2021. Kim H et al. Published online at http://www.aginganddisease.org/EN/10.14336/AD.2021.0621

SUPPLEMENTARY DATA

hsa-miR-128-3p	68	45	-0.61	8.3960
hsa-miR-140-3p_L-1R+2	68	308	2.17	9.6115
hsa-miR-98-5p	64	34	-0.91	10.1471
hsa-miR-148b-3p	64	613	3.26	45.9636
hsa-miR-193b-3p	64	963	3.91	94.5264
hsa-miR-423-5p	60	951	3.99	95.8464
hsa-miR-505-3p_L-1R+2	56	1,160	4.38	130.4698
hsa-miR-302d-3p	54	0	-inf	26.1580
hsa-miR-133a-3p_L-1R+1	51	14	-1.83	13.2728
hsa-let-7c-5p	44	135	1.61	1.6857
hsa-miR-15a-5p	43	12	-1.86	11.1943
hsa-let-7d-3p	43	652	3.93	64.4184
hsa-miR-92b-3p	43	662	3.95	65.7766
hsa-miR-196b-5p	43	0	-inf	20.8279
hsa-miR-499a-5p	43	0	-inf	20.8279
mdo-miR-22-3p	39	6	-2.66	12.9814
hsa-miR-340-5p	39	23	-0.76	5.7285
hsa-miR-4286_R+1	39	97	1.33	0.4428
hsa-miR-106b-5p	34	6	-2.59	10.8814
hsa-miR-210-3p	34	39	0.20	1.7666
hsa-miR-21-3p	34	77	1.18	0.1635
hsa-miR-214-5p	34	194	2.50	8.9391
mdo-miR-210-5p_L+1R-1	34	0	-inf	16.4679
hsa-miR-31-3p_R+1	30	4	-2.93	10.5260
mmu-miR-3968_L-3_1ss14AT	30	14	-1.15	5.6511
hsa-miR-361-5p	30	16	-0.93	5.0072
mmu-mir-6240-p3_1ss10TG	30	103	1.79	1.9095
mmu-mir-6240-p3	30	110	1.88	2.4184
hsa-miR-744-5p_R-1	30	183	2.61	9.1432
hsa-miR-197-3p	30	388	3.70	35.4397
hsa-miR-208a-3p	30	0	-inf	14.5300
PC-5p-480_1295	30	0	-inf	14.5300
PC-3p-43921_7	30	0	-inf	14.5300
hsa-miR-29b-3p	28	3	-3.19	10.4039
hsa-miR-374b-5p	26	12	-1.12	4.8907
hsa-miR-146b-5p_R+1	26	17	-0.63	3.6164
hsa-miR-155-5p_R-1	26	24	-0.12	2.2180
hsa-miR-185-5p	26	28	0.14	1.7001
sha-miR-125a_R+2	26	44	0.78	0.3504
hsa-miR-432-5p	26	690	4.75	84.3144
hsa-miR-208b-3p	26	0	-inf	12.5924
oan-miR-15b-5p	26	0	-inf	12.5924
PC-5p-51899_6	26	0	-inf	12.5924
mmu-mir-6236-p3_1ss2AG	24	69	1.56	0.7342
hsa-miR-4521_R+4	21	2	-3.29	8.0932
hsa-miR-18a-5p	21	3	-2.81	7.3528
hsa-miR-137-3p	21	11	-0.97	3.7124
hsa-miR-140-5p	21	16	-0.44	2.5648
hsa-miR-425-5p_R+2	21	23	0.09	1.2814
hsa-let-7i-3p	21	50	1.22	0.2113
bta-miR-2478_L-2	21	58	1.43	0.5515
mmu-mir-6240-p5	21	115	2.42	5.2250
hsa-miR-874-3p_R+1	21	151	2.82	9.0774
hsa-miR-138-5p	19	69	1.83	1.5198
hsa-miR-107_R-2	11	12	0.08	0.7438
hsa-miR-450a-5p	11	6	-0.86	2.0898
bta-miR-11987_L-1_1ss8TA	11	12	0.11	0.7438
hsa-miR-302a-3p_R-2_1ss19GA	7	0	-inf	3.3899
hsa-miR-302b-3p_R-2	7	0	-inf	3.3899

© 2021. Kim H et al. Published online at http://www.aginganddisease.org/EN/10.14336/AD.2021.0621

SUPPLEMENTARY DATA

hsa-miR-320c_R-1	4	36	3.09	2.9255
hsa-miR-320b_R-2	4	103	4.59	12.7495
hsa-miR-370-3p	0	1,531	inf	265.5173
bta-miR-2904_R+3_1ss1GC	0	1,121	inf	194.0689
hsa-miR-654-3p_R-2	0	1,066	inf	184.4567
bta-mir-2904-2-p3	0	974	inf	168.4763
hsa-miR-149-5p	0	842	inf	145.6704
hsa-miR-409-3p	0	601	inf	103.7564
hsa-miR-28-3p	0	475	inf	81.9217
mmu-mir-6236-p5_1ss20GC	0	443	inf	76.4694
hsa-miR-493-5p	0	393	inf	67.8290
mmu-mir-6236-p5_1ss26GC	0	386	inf	66.4488
hsa-miR-328-3p	0	373	inf	64.1621
hsa-miR-1296-5p	0	329	inf	56.7090
mmu-mir-6236-p5_1ss25GC	0	323	inf	55.5462
hsa-miR-339-5p_R-1	0	252	inf	43.2968
hsa-miR-381-3p	0	252	inf	43.2968
hsa-miR-433-3p	0	239	inf	41.0145
mmu-mir-6236-p3	0	228	inf	39.2274
hsa-miR-134-5p	0	221	inf	38.0810
hsa-miR-485-3p	0	203	inf	34.9151
hsa-miR-345-5p	0	201	inf	34.4232
hsa-miR-1307-3p_R+1	0	186	inf	31.9640
hsa-miR-486-5p	0	175	inf	30.1347
hsa-miR-150-5p	0	159	inf	27.1566
hsa-miR-451a_R-1	0	154	inf	26.5042
hsa-miR-887-3p_R+2	0	150	inf	25.8195
hsa-miR-10a-5p_R-1	0	150	inf	25.8194
ssc-mir-1285-p3_1ss24TA	0	137	inf	23.5576
mmu-mir-6240-p5_1ss12AG_2	0	131	inf	22.4253
mmu-mir-6240-p3_1ss12AG	0	131	inf	22.4253
hsa-miR-126-3p_1ss22GA	0	130	inf	22.1741
hsa-miR-378a-3p	0	128	inf	21.9755
hsa-miR-543	0	125	inf	21.5039
hsa-miR-223-3p	0	118	inf	20.1463
hsa-miR-431-3p	0	117	inf	19.8862
hsa-miR-224-5p_L-1R-2	0	117	inf	19.8862
hsa-miR-409-5p	0	117	inf	19.8862
hsa-miR-487b-3p	0	115	inf	19.7061
bta-mir-2887-1-p5_1ss15AT	0	108	inf	18.5578
bta-mir-2887-1-p3_1ss15AT	0	108	inf	18.5578
hsa-miR-940	0	109	inf	18.5379
hsa-miR-145-3p	0	109	inf	18.5379
hsa-miR-125b-1-3p_R-1_1ss21CT	0	106	inf	18.1121
bta-mir-2887-1-p3_1ss11AT	0	105	inf	17.8627
hsa-miR-1271-5p	0	100	inf	17.1876
hsa-miR-503-5p_R-1	0	98	inf	16.7526
hsa-miR-342-3p_R+1	0	97	inf	16.5099
mmu-mir-6240-p3_1ss25GT	0	97	inf	16.5099
hsa-miR-493-3p	0	97	inf	16.5099
hsa-miR-665_R-2	0	97	inf	16.5099
hsa-miR-574-5p	0	89	inf	15.1543
mmu-mir-6240-p5_1ss12AG_1	0	87	inf	14.9257
hsa-miR-106b-3p	0	88	inf	14.8989
hsa-miR-93-3p_R+1	0	86	inf	14.7087
mmu-mir-6236-p3_1ss22GC	0	80	inf	13.5471
hsa-miR-331-3p_R+1	0	75	inf	12.8702
hsa-miR-424-3p	0	75	inf	12.8702
hsa-miR-136-3p	0	73	inf	12.4348

© 2021. Kim H et al. Published online at http://www.aginganddisease.org/EN/10.14336/AD.2021.0621

hsa-miR-1260b_1ss9AG	0	68	inf	11.5137
hsa-miR-656-3p	0	67	inf	11.2551
hsa-miR-200b-3p_R+1	0	65	inf	11.0713
hsa-miR-324-3p_L-3R+1	0	64	inf	10.8344
hsa-miR-151a-3p	0	61	inf	10.3887
hsa-miR-329-3p	0	61	inf	10.3887
hsa-miR-130b-5p_R+1	0	58	inf	9.9214
hsa-miR-487a-5p	0	58	inf	9.9214
mmu-mir-6236-p3_1ss8AG	0	56	inf	9.4737
hsa-miR-1180-3p	0	55	inf	9.2239
hsa-miR-99b-3p_R+1	0	52	inf	8.7923
hsa-miR-3615_R+2	0	50	inf	8.5496
mmu-mir-6240-p5_1ss22GT	0	49	inf	8.3374
mmu-mir-6240-p3_1ss19GT	0	49	inf	8.3374
mmu-mir-6240-p5_1ss13AG	0	48	inf	8.1101
hsa-miR-671-3p	0	48	inf	8.1101
bta-mir-2887-1-p5_1ss21AT	0	45	inf	7.6525
hsa-miR-323a-3p_L+1	0	45	inf	7.6525
hsa-miR-330-3p	0	45	inf	7.6525
hsa-miR-1307-5p_R+2	0	46	inf	7.6022
hsa-miR-500a-3p	0	46	inf	7.6022
hsa-miR-889-3p	0	44	inf	7.4272
hsa-miR-425-3p	0	43	inf	7.1848
hsa-miR-154-5p	0	43	inf	7.1848
hsa-miR-377-5p	0	41	inf	6.9670
hsa-miR-122-5p_R-1	0	42	inf	6.9242
hsa-miR-1185-1-3p	0	39	inf	6.5032
hsa-miR-221-5p_R+1	0	39	inf	6.5032
hsa-miR-369-3p	0	37	inf	6.2810
mmu-miR-1983	0	38	inf	6.2449
hsa-miR-589-5p_R-1	0	38	inf	6.2449
hsa-miR-410-3p	0	36	inf	6.0591
hsa-miR-483-3p_L-1R+2	0	36	inf	6.0590
hsa-miR-132-3p	0	35	inf	5.8206
hsa-miR-323b-3p_R-1	0	35	inf	5.8206
hsa-miR-24-2-5p_L+1	0	33	inf	5.5944
hsa-miR-182-5p_R+1_1ss24TC	0	33	inf	5.5944
mmu-mir-6236-p5_1ss8CG	0	33	inf	5.5944
bta-miR-150_1ss9CT	0	33	inf	5.5943
hsa-miR-337-3p_L-1	0	34	inf	5.5644
hsa-miR-652-3p_R+1	0	34	inf	5.5644
hsa-miR-758-3p_R-1	0	34	inf	5.5644
hsa-miR-193b-5p	0	32	inf	5.3737
hsa-miR-339-3p	0	32	inf	5.3737
hsa-miR-184_R-2	0	31	inf	5.1368
hsa-miR-10401-3p_L+1R-1	0	31	inf	5.1368
bta-mir-2887-1-p5_1ss18AT	0	31	inf	5.1368
hsa-let-7b-3p_1ss22CT	0	29	inf	4.9070
hsa-miR-125b-2-3p_L-2R+2	0	29	inf	4.9070
hsa-miR-183-5p_L-1R+1	0	29	inf	4.9070
hsa-miR-7706_R-1	0	30	inf	4.8825
pal-miR-9993a-3p_1ss11GA	0	30	inf	4.8825
hsa-mir-1285-1-p5	0	28	inf	4.6873
hsa-miR-323a-5p	0	28	inf	4.6873
hsa-miR-3117-3p_L-1R+2_1ss4GA	0	28	inf	4.6873
hsa-miR-3200-3p_R+1	0	28	inf	4.6873
hsa-miR-487b-5p	0	25	inf	4.2188
hsa-miR-142-3p_L-1	0	26	inf	4.1989
hsa-miR-615-3p	0	26	inf	4.1989

© 2021. Kim H et al. Published online at http://www.aginganddisease.org/EN/10.14336/AD.2021.0621

hsa-miR-539-3p	0	24	inf	3.9997
hsa-miR-618	0	23	inf	3.7649
hsa-miR-769-5p	0	23	inf	3.7649
hsa-miR-7977_1ss6AG	0	23	inf	3.7649
hsa-miR-139-5p	0	23	inf	3.7649
hsa-miR-369-5p_R-1	0	21	inf	3.5297
mmu-miR-146a-5p_R+1	0	21	inf	3.5297
hsa-miR-760_R+2	0	22	inf	3.5133
eca-mir-8986a-p5_1ss1GA	0	22	inf	3.5133
hsa-miR-9-5p	0	20	inf	3.3107
hsa-miR-361-3p	0	20	inf	3.3106
bta-miR-150	0	20	inf	3.3106
hsa-miR-455-3p_R+1	0	19	inf	3.0760
hsa-miR-7704_R-1	0	19	inf	3.0760
ssc-mir-1285-p5	0	19	inf	3.0760
bta-miR-4286_R+2	0	19	inf	3.0760
hsa-let-7e-3p	0	19	inf	3.0760
hsa-miR-377-3p	0	19	inf	3.0760
hsa-miR-532-5p	0	17	inf	2.8392
hsa-miR-655-3p	0	17	inf	2.8392
hsa-miR-1306-5p	0	17	inf	2.8392
hsa-miR-1343-3p	0	17	inf	2.8392
hsa-miR-296-5p	0	17	inf	2.8392
hsa-miR-668-3p_R+1	0	17	inf	2.8392
mmu-miR-7689-3p	0	17	inf	2.8392
hsa-miR-125a-3p_R-1	0	18	inf	2.8251
cgr-miR-1260_R+4	0	18	inf	2.8251
hsa-miR-16-2-3p_L+1R-1	0	18	inf	2.8251
hsa-miR-34c-5p	0	18	inf	2.8251
hsa-miR-485-5p_R+1	0	16	inf	2.6194
hsa-miR-2110_R-1	0	16	inf	2.6194
hsa-miR-671-5p_R+1	0	16	inf	2.6194
hsa-miR-130b-3p	0	15	inf	2.3841
hsa-miR-185-3p_R-1	0	15	inf	2.3841
oan-let-7e-5p	0	15	inf	2.3841
cgr-miR-1260_R+2_3	0	15	inf	2.3841
cgr-miR-1260_R+2_2	0	15	inf	2.3841
cgr-miR-1260_R+2_1	0	15	inf	2.3841
ssc-mir-4332-p5_1ss16CA	0	13	inf	2.1466
ssc-mir-4332-p5_1ss12CA	0	13	inf	2.1466
hsa-miR-22-5p	0	13	inf	2.1466
hsa-miR-299-3p	0	13	inf	2.1466
hsa-miR-532-3p	0	13	inf	2.1466
hsa-miR-877-5p_R+2	0	13	inf	2.1466
mmu-mir-1983-p5_1ss1GA	0	13	inf	2.1466
hsa-miR-502-3p_R+1	0	14	inf	2.1332
hsa-miR-941	0	14	inf	2.1332
hsa-miR-99a-3p_R+1	0	14	inf	2.1332
hsa-miR-365b-5p_L+1	0	14	inf	2.1332
hsa-let-7a-3p_R+1	0	12	inf	1.9249
hsa-miR-224-3p_L-1	0	12	inf	1.9249
cja-mir-3135-p3_1ss11CA	0	12	inf	1.9249
hsa-miR-501-3p	0	12	inf	1.9249
hsa-miR-675-3p_R+2	0	12	inf	1.9249
mmu-mir-6236-p5_1ss3CG	0	12	inf	1.9249
hsa-miR-27a-5p	0	11	inf	1.6876
hsa-miR-494-3p_R+1	0	11	inf	1.6876
hsa-miR-3187-3p	0	11	inf	1.6876
mml-miR-7170-3p_L+2	0	11	inf	1.6876

© 2021. Kim H et al. Published online at http://www.aginganddisease.org/EN/10.14336/AD.2021.0621

PC-5p-41351_8	0	11	inf	1.6876
hsa-miR-30a-3p	0	11	inf	1.6876
hsa-miR-129-5p	0	11	inf	1.6876
hsa-miR-337-5p_R-1	0	11	inf	1.6876
hsa-miR-382-5p	0	11	inf	1.6876
hsa-miR-450b-5p_R-1	0	11	inf	1.6876
hsa-miR-654-5p	0	11	inf	1.6876
hsa-miR-1468-5p_R+1	0	11	inf	1.6876
bta-miR-339a_R+1	0	11	inf	1.6876
cgr-miR-1260_R+1	0	11	inf	1.6876
hsa-miR-138-1-3p_L+2R-1	0	9	inf	1.4503
hsa-miR-192-5p	0	9	inf	1.4503
hsa-miR-194-5p	0	9	inf	1.4503
hsa-miR-365a-5p_R-1	0	9	inf	1.4503
hsa-miR-1185-2-3p	0	9	inf	1.4503
hsa-miR-1303	0	9	inf	1.4503
mmu-miR-326-3p	0	9	inf	1.4503
PC-5p-20333_21	0	9	inf	1.4503
PC-3p-30410_12	0	9	inf	1.4503
hsa-miR-18a-3p	0	9	inf	1.4503
hsa-miR-92b-5p_R+2	0	9	inf	1.4503
hsa-miR-411-5p	0	9	inf	1.4503
hsa-miR-1260a_R+3_1ss9TG	0	9	inf	1.4503
hsa-miR-2277-5p_R-1	0	9	inf	1.4503
hsa-miR-4746-5p	0	9	inf	1.4503
mmu-let-7j_1ss8TG	0	9	inf	1.4503
hsa-miR-200a-3p_R+1	0	10	inf	1.4356
hsa-miR-324-5p_R+1	0	10	inf	1.4356
cja-mir-367-p3	0	10	inf	1.4356
PC-3p-44144_7	0	10	inf	1.4356
hsa-miR-205-5p_R-3	0	10	inf	1.4356
mml-miR-218-1-3p_L+4R-2	0	10	inf	1.4356
hsa-miR-28-5p	0	8	inf	1.2242
hsa-miR-126-5p	0	8	inf	1.2242
hsa-miR-144-5p	0	8	inf	1.2242
hsa-miR-320d_R-1	0	8	inf	1.2242
hsa-miR-3909	0	8	inf	1.2242
mmu-miR-155-5p_R+1	0	8	inf	1.2242
hsa-miR-181a-2-3p	0	8	inf	1.2242
hsa-miR-4326_R+4	0	8	inf	1.2242
hsa-miR-6511a-3p	0	8	inf	1.2242
cgr-mir-1285-p3_1ss2TC	0	8	inf	1.2242
hsa-miR-23a-5p_R-1	0	7	inf	0.9823
hsa-miR-30e-3p_1ss22CT	0	7	inf	0.9823
hsa-miR-203a-3p_R+1	0	7	inf	0.9823
hsa-miR-766-3p	0	7	inf	0.9823
hsa-mir-770-p3_1ss23CT	0	7	inf	0.9823
cgr-mir-1285-p5_1ss7TC	0	7	inf	0.9823
hsa-miR-15b-3p_R-1	0	7	inf	0.9823
hsa-miR-127-5p	0	7	inf	0.9823
hsa-miR-383-5p	0	7	inf	0.9823
hsa-miR-509-3-5p	0	7	inf	0.9823
hsa-mir-619-p5_1ss2GT	0	7	inf	0.9823
hsa-mir-7851-p5_1ss12TG	0	7	inf	0.9823
hsa-miR-32-5p_R-1	0	5	inf	0.7453
hsa-miR-3679-5p	0	5	inf	0.7453
hsa-miR-12136_R+8	0	5	inf	0.7453
bta-mir-2887-1-p3_1ss5CA	0	5	inf	0.7453
PC-3p-29366_13	0	5	inf	0.7453

© 2021. Kim H et al. Published online at http://www.aginganddisease.org/EN/10.14336/AD.2021.0621

PC-5p-29949_12	0	5	inf	0.7453
hsa-miR-210-5p	0	5	inf	0.7453
hsa-miR-380-3p_R-1	0	5	inf	0.7453
hsa-miR-411-3p_R-1	0	5	inf	0.7453
hsa-miR-2277-3p_R+1	0	5	inf	0.7453
PC-3p-32430_11	0	5	inf	0.7453
PC-3p-33077_11	0	5	inf	0.7453
PC-5p-33194_11	0	5	inf	0.7453
hsa-miR-129-2-3p	0	6	inf	0.7281
hsa-miR-144-3p	0	6	inf	0.7281
hsa-miR-496_R-1	0	6	inf	0.7281
hsa-miR-1249-3p	0	6	inf	0.7281
hsa-miR-1301-3p	0	6	inf	0.7281
hsa-miR-1304-3p_1ss13CA	0	6	inf	0.7281
hsa-miR-2682-3p_L+1R-1_1ss22CT	0	6	inf	0.7281
hsa-miR-3605-3p	0	6	inf	0.7281
hsa-miR-4725-3p_R+1	0	6	inf	0.7281
hsa-miR-9901_L+4R-2	0	6	inf	0.7281
ggo-mir-1291-p3	0	6	inf	0.7281
mmu-mir-3535-p3	0	6	inf	0.7281
mmu-mir-6240-p5_1ss1GA	0	6	inf	0.7281
bta-miR-296-3p_L-1R+1	0	6	inf	0.7281
PC-3p-17170_26	0	6	inf	0.7281
PC-3p-33154_11	0	6	inf	0.7281
PC-5p-28757_13	0	6	inf	0.7281
hsa-miR-34c-3p	0	4	inf	0.5081
hsa-miR-151b_R+2	0	4	inf	0.5081
hsa-miR-181c-5p_R+1	0	4	inf	0.5081
hsa-miR-217-5p	0	4	inf	0.5081
hsa-miR-494-5p_R-1	0	4	inf	0.5081
hsa-miR-1226-3p	0	4	inf	0.5081
hsa-miR-1290_1ss13TG	0	4	inf	0.5081
hsa-miR-3940-3p	0	4	inf	0.5081
hsa-mir-5684-p3_1ss19CG	0	4	inf	0.5081
hsa-miR-6511b-3p_R+1	0	4	inf	0.5081
hsa-mir-8086-p3_1ss19AG	0	4	inf	0.5081
hsa-miR-10527-5p_R-1	0	4	inf	0.5081
ptr-mir-4743-p3_1ss23GT	0	4	inf	0.5081
cgr-mir-1260-p3_1ss1AG	0	4	inf	0.5081
mmu-mir-6236-p3_1ss1CG	0	4	inf	0.5081
bta-miR-7857-3p_R-1	0	4	inf	0.5081
PC-3p-33979_10	0	4	inf	0.5081
PC-3p-33419_10	0	4	inf	0.5081
hsa-let-7a-2-3p	0	4	inf	0.5081
hsa-miR-136-5p_R-1	0	4	inf	0.5081
hsa-miR-421	0	4	inf	0.5081
hsa-miR-452-5p_R+1	0	4	inf	0.5081
hsa-miR-501-5p_R+2	0	4	inf	0.5081
hsa-miR-664b-3p	0	4	inf	0.5081
hsa-miR-942-5p_R+1	0	4	inf	0.5081
hsa-mir-1285-1-p3_1ss19TC	0	4	inf	0.5081
hsa-miR-2355-3p_L-2R+1	0	4	inf	0.5081
hsa-miR-3154	0	4	inf	0.5081
eca-miR-9182_L-1R+2	0	4	inf	0.5081
oan-miR-1386	0	4	inf	0.5081
pal-miR-9993b-3p	0	4	inf	0.5081
oan-miR-458-3p	0	4	inf	0.5081
PC-3p-37780_9	0	4	inf	0.5081
PC-5p-35952_9	0	4	inf	0.5081

© 2021. Kim H et al. Published online at http://www.aginganddisease.org/EN/10.14336/AD.2021.0621

SUPPLEMENTARY DATA

PC-3p-35644_9	0	4	inf	0.5081
PC-3p-35774_9	0	4	inf	0.5081
PC-3p-36658_9	0	4	inf	0.5081
PC-5p-36216_9	0	4	inf	0.5081
PC-3p-36756_9	0	4	inf	0.5081
PC-5p-36968_9	0	4	inf	0.5081
hsa-miR-23b-5p	0	3	inf	0.2552
hsa-miR-96-5p	0	3	inf	0.2552
hsa-miR-98-3p_L-1R+1_1ss22CT	0	3	inf	0.2552
hsa-miR-200c-3p	0	3	inf	0.2552
hsa-miR-212-5p	0	3	inf	0.2552
hsa-miR-378a-5p	0	3	inf	0.2552
hsa-miR-487a-3p	0	3	inf	0.2552
hsa-miR-541-5p_R-4	0	3	inf	0.2552
hsa-miR-770-5p_R-1	0	3	inf	0.2552
hsa-miR-1180-5p_R-1	0	3	inf	0.2552
hsa-miR-1229-3p	0	3	inf	0.2552
hsa-miR-1234-3p_R+1	0	3	inf	0.2552
hsa-miR-1306-3p_L+1R+3	0	3	inf	0.2552
hsa-miR-10400-5p_R-3_1ss12GA	0	3	inf	0.2552
eca-miR-8986a_L-1R-1	0	3	inf	0.2552
ssc-miR-30a-3p_L-1R+1	0	3	inf	0.2552
oan-miR-1388-3p	0	3	inf	0.2552
PC-3p-41871_8	0	3	inf	0.2552
PC-3p-42880_8	0	3	inf	0.2552
PC-3p-39587_8	0	3	inf	0.2552
PC-3p-39792_8	0	3	inf	0.2552
PC-3p-39271_8	0	3	inf	0.2552
PC-5p-40074_8	0	3	inf	0.2552
hsa-miR-7-5p	0	3	inf	0.2552
hsa-miR-25-5p_R+2	0	3	inf	0.2552
hsa-miR-30c-2-3p	0	3	inf	0.2552
hsa-miR-103a-2-5p_R-1	0	3	inf	0.2552
hsa-miR-146b-3p_L+1R-1	0	3	inf	0.2552
hsa-miR-152-5p	0	3	inf	0.2552
hsa-miR-181d-5p_R+1	0	3	inf	0.2552
hsa-miR-181a-3p	0	3	inf	0.2552
hsa-miR-202-5p_R-1	0	3	inf	0.2552
hsa-miR-204-5p	0	3	inf	0.2552
hsa-miR-212-3p	0	3	inf	0.2552
hsa-miR-215-5p_1ss20AC	0	3	inf	0.2552
hsa-miR-219a-5p	0	3	inf	0.2552
hsa-miR-299-5p_R-1	0	3	inf	0.2552
hsa-miR-375-3p	0	3	inf	0.2552
hsa-miR-455-5p	0	3	inf	0.2552
hsa-miR-483-5p	0	3	inf	0.2552
mmu-miR-496a-5p_L-1R+2_1ss7CT	0	3	inf	0.2552
hsa-miR-506-3p_L+1	0	3	inf	0.2552
hsa-miR-589-3p_1ss24AC	0	3	inf	0.2552
hsa-miR-625-3p	0	3	inf	0.2552
hsa-miR-769-3p_R-1	0	3	inf	0.2552
hsa-miR-1197	0	3	inf	0.2552
hsa-miR-1269a	0	3	inf	0.2552
hsa-mir-1292-p5	0	3	inf	0.2552
hsa-miR-1908-5p	0	3	inf	0.2552
hsa-miR-1910-5p_R+1	0	3	inf	0.2552
hsa-miR-3127-3p_R-1	0	3	inf	0.2552
hsa-mir-3135b-p3_1ss3AG	0	3	inf	0.2552
hsa-miR-3195_L+3R-1	0	3	inf	0.2552

© 2021. Kim H et al. Published online at http://www.aginganddisease.org/EN/10.14336/AD.2021.0621

hsa-miR-3661	0	3	inf	0.2552
hsa-miR-3934-5p_R-2	0	3	inf	0.2552
hsa-miR-4454_1ss2GA	0	3	inf	0.2552
hsa-mir-6132-p3	0	3	inf	0.2552
hsa-miR-6735-5p_R-2	0	3	inf	0.2552
hsa-miR-6858-3p	0	3	inf	0.2552
hsa-miR-6881-5p	0	3	inf	0.2552
hsa-miR-6882-3p_R-2_1ss22CT	0	3	inf	0.2552
mmu-miR-1839-5p	0	3	inf	0.2552
cfa-mir-8903-p3_1ss9AG	0	3	inf	0.2552
oan-miR-27b-3p_R+1	0	3	inf	0.2552
mdo-miR-210-3p_L-1R+1	0	3	inf	0.2552
bta-mir-1246-p3_1ss17AG	0	3	inf	0.2552
mmu-mir-3535-p5	0	3	inf	0.2552
oan-miR-15c-5p_R+1_1ss1TC	0	3	inf	0.2552
eca-miR-451_R-1_1ss21TG	0	3	inf	0.2552
mmu-miR-5099_L+2R-1	0	3	inf	0.2552
PC-5p-44344_7	0	3	inf	0.2552
PC-5p-44472_7	0	3	inf	0.2552
PC-3p-23151_18	0	3	inf	0.2552
PC-5p-48417_7	0	3	inf	0.2552
PC-5p-47000_7	0	3	inf	0.2552
PC-3p-48386_7	0	3	inf	0.2552
PC-5p-27475_14	0	3	inf	0.2552
PC-3p-47110_7	0	3	inf	0.2552
hsa-miR-7-1-3p_L-1R+1	0	2	inf	0.0000
hsa-miR-29c-3p_R-1	0	2	inf	0.0000
hsa-miR-124-3p_R-1	0	2	inf	0.0000
hsa-miR-191-3p_L-1_1ss19CT	0	2	inf	0.0000
hsa-miR-216a-5p	0	2	inf	0.0000
hsa-miR-378c_R-5	0	2	inf	0.0000
hsa-miR-495-3p	0	2	inf	0.0000
hsa-miR-497-5p_R+1	0	2	inf	0.0000
hsa-miR-509-3p_R+1	0	2	inf	0.0000
rno-miR-544-5p_L+1	0	2	inf	0.0000
hsa-miR-548aq-3p_R-1	0	1	inf	0.0000
hsa-miR-550a-3p_L-1R+1	0	2	inf	0.0000
hsa-miR-550a-5p_R-2	0	1	inf	0.0000
hsa-miR-561-5p	0	2	inf	0.0000
hsa-miR-584-5p_R-1	0	2	inf	0.0000
hsa-miR-660-5p_R+1	0	2	inf	0.0000
hsa-miR-675-5p_R-1	0	2	inf	0.0000
hsa-miR-1185-5p	0	2	inf	0.0000
hsa-miR-1268b_R-2	0	2	inf	0.0000
hsa-miR-1268a	0	2	inf	0.0000
hsa-miR-1843_R+2	0	2	inf	0.0000
hsa-mir-2110-p3	0	2	inf	0.0000
hsa-miR-3124-5p_R+1	0	2	inf	0.0000
hsa-mir-3155a-p5_1ss23AT	0	2	inf	0.0000
hsa-miR-3158-3p	0	1	inf	0.0000
hsa-miR-3177-3p	0	2	inf	0.0000
hsa-mir-3648-1-p5	0	2	inf	0.0000
hsa-miR-3651	0	2	inf	0.0000
hsa-mir-3909-p5_1ss21GT	0	2	inf	0.0000
hsa-miR-3944-3p	0	2	inf	0.0000
hsa-miR-4254_R-1	0	2	inf	0.0000
hsa-miR-4449_R+1	0	2	inf	0.0000
hsa-mir-4477b-p5_1ss9GA	0	2	inf	0.0000
hsa-mir-4477a-p5_1ss11CG	0	2	inf	0.0000

© 2021. Kim H et al. Published online at http://www.aginganddisease.org/EN/10.14336/AD.2021.0621

hsa-miR-6785-5p_L+1R+1	0	2	inf	0.0000
hsa-miR-7976_R+3	0	2	inf	0.0000
pal-miR-9298-5p_R-5	0	2	inf	0.0000
bta-miR-20b_R+1	0	1	inf	0.0000
eca-mir-8986b-p5_1ss1CG	0	2	inf	0.0000
ssc-miR-339_R+1	0	2	inf	0.0000
bta-mir-1246-p5_1ss18AG	0	1	inf	0.0000
mmu-miR-2137_R-1_1ss16AG	0	2	inf	0.0000
mmu-miR-2137_L-2_1ss16AG	0	2	inf	0.0000
bta-miR-20b_1ss19GA	0	2	inf	0.0000
mdo-miR-210-3p_L-1R+2	0	2	inf	0.0000
bta-miR-429	0	2	inf	0.0000
oan-miR-1388-5p	0	1	inf	0.0000
bta-miR-1388-5p_R+1	0	1	inf	0.0000
mmu-miR-8117_L-1R-1_1ss15GA	0	2	inf	0.0000

Supplementary Table 5. KEGG pathway ranking summary of predicted targets the top 6 ranked miRNAs in EVs from early-passage iMSCs.

TERM	TOTAL_GENES_OF_THE_TERM	UNION_TARGETS_IN_THE_TERM	MIRS_IN_THE_TERM	SCORE
MAPK_SIGNALING_PATHWAY	272	80	6	6.151
TGF-BETA_SIGNALING_PATHWAY	84	35	6	5.35
CYTOKINE-CYTOKINE_RECEPTOR_INTERACTION	275	53	6	4.739
PATHWAYS_IN_CANCER	325	92	6	4.238
REGULATION_OF_ACTIN_CYTOSKELETON	213	64	6	3.889
P53_SIGNALING_PATHWAY	68	22	6	3.683
PANCREATIC_CANCER	70	23	6	3.67
CHAGAS_DISEASE	104	29	6	3.519
NEUROTROPHIN_SIGNALING_PATHWAY	127	42	6	3.486
MELANOMA	71	24	6	3.435
AXON_GUIDANCE	129	49	6	2.902
SMALL_CELL_LUNG_CANCER	84	24	6	2.888
CHRONIC_MYELOID_LEUKEMIA	73	21	6	2.884
GLIOMA	65	23	6	2.784
COLORECTAL_CANCER	62	17	6	2.699
FOCAL_ADHESION	199	57	6	2.681
JAK-STAT_SIGNALING_PATHWAY	155	29	6	2.652
PROSTATE_CANCER	89	26	6	2.494
ENDOCYTOSIS	201	56	6	2.462
APOPTOSIS	88	19	6	2.438

© 2021. Kim H et al. Published online at http://www.aginganddisease.org/EN/10.14336/AD.2021.0621

AMOEBIASIS	105	25	6	2.33<br>4
CHEMOKINE_SIGNALING_PATHWAY	189	36	6	2.31
T_CELL_RECEPTOR_SIGNALING_PATHWAY	108	36	6	2.15<br>4
CELL_CYCLE	124	29	6	2.08<br>3
PROTEIN_PROCESSING_IN_ENDOPLASMIC_RETICULUM	166	42	6	2.01<br>7
TOXOPLASMOSIS	132	24	6	1.99<br>2
UBIQUITIN_MEDIATED_PROTEOLYSIS	135	40	6	1.99<br>1
ECM-RECEPTOR_INTERACTION	84	28	6	1.99
AMYOTROPHIC_LATERAL_SCLEROSIS_(ALS)	54	18	6	1.95<br>4
WNT_SIGNALING_PATHWAY	150	39	6	1.93<br>4
GNRH_SIGNALING_PATHWAY	101	29	6	1.87<br>8
ERBB_SIGNALING_PATHWAY	87	31	6	1.86<br>3
LONG-TERM_POTENTIATION	70	24	6	1.85<br>9
BLADDER_CANCER	42	15	6	1.81<br>5
NON-SMALL_CELL_LUNG_CANCER	54	17	6	1.68<br>4
MALARIA	51	10	4	1.67<br>3
DILATED_CARDIOMYOPATHY	90	27	6	1.61<br>2
FC_GAMMA_R-MEDIATED_PHAGOCYTOSIS	94	28	6	1.60<br>6
OOCYTE_MEIOSIS	112	35	6	1.57
HYPERTROPHIC_CARDIOMYOPATHY_(HCM)	87	24	6	1.53<br>2
TOLL-LIKE_RECEPTOR_SIGNALING_PATHWAY	102	19	6	1.51<br>3
INSULIN_SIGNALING_PATHWAY	137	35	6	1.49<br>7
CALCIUM_SIGNALING_PATHWAY	177	43	6	1.47
PROGESTERONE-MEDIATED_OOCYTE_MATURATION	86	23	6	1.38<br>1
VASCULAR_SMOOTH_MUSCLE_CONTRACTION	126	28	6	1.37<br>7
SHIGELLOSIS	61	13	6	1.36<br>3
VEGF_SIGNALING_PATHWAY	76	20	6	1.34<br>1
LEISHMANIASIS	72	15	6	1.32<br>1
ADHERENS_JUNCTION	73	22	6	1.29<br>5
ARRHYTHMOGENIC_RIGHT_VENTRICULAR_CARDIOMYOPATHY_(ARVC)	74	19	6	1.29
B_CELL_RECEPTOR_SIGNALING_PATHWAY	75	16	5	1.28<br>7
PROTEIN_DIGESTION_AND_ABSORPTION	80	17	6	1.28<br>7

© 2021. Kim H et al. Published online at http://www.aginganddisease.org/EN/10.14336/AD.2021.0621

TYPE_I_DIABETES_MELLITUS	43	7	3	1.281
HEPATITIS_C	134	27	6	1.255
FC_EPSILON_RI_SIGNALING_PATHWAY	79	21	6	1.247
NATURAL_KILLER_CELL_MEDIATED_CYTOTOXICITY	140	21	5	1.226
PHOSPHATIDYLINOSITOL_SIGNALING_SYSTEM	78	18	6	1.208
MELANOGENESIS	101	27	6	1.2
ADIPOCYTOKINE_SIGNALING_PATHWAY	68	13	5	1.175
NOTCH_SIGNALING_PATHWAY	47	16	6	1.137
GALACTOSE_METABOLISM	26	5	3	1.132
PRION_DISEASES	36	10	6	1.099
RENAL_CELL_CARCINOMA	70	21	6	1.056
CELL_ADHESION_MOLECULES_(CAMS)	133	27	6	1.047
GAP_JUNCTION	90	23	6	1.043
TIGHT_JUNCTION	132	25	6	1.018
TYPE_II_DIABETES_MELLITUS	47	12	6	1.018
ALZHEIMER'S_DISEASE	168	27	6	1.013
GASTRIC_ACID_SECRETION	74	22	6	1.01
MTOR_SIGNALING_PATHWAY	52	18	6	1.01
ALLOGRAFT_REJECTION	37	6	3	0.976
NOD-LIKE_RECEPTOR_SIGNALING_PATHWAY	62	9	5	0.96
BACTERIAL_INVASION_OF_EPITHELIAL_CELLS	70	17	4	0.942
ALDOSTERONE-REGULATED_SODIUM_REABSORPTION	42	17	6	0.94
ENDOMETRIAL_CANCER	52	13	6	0.929
NEUROACTIVE_LIGAND-RECEPTOR_INTERACTION	318	45	6	0.922
RIG-I-LIKE_RECEPTOR_SIGNALING_PATHWAY	71	12	6	0.907
INOSITOL_PHOSPHATE_METABOLISM	57	11	5	0.898
GRAFT-VERSUS-HOST_DISEASE	41	5	3	0.894
EPITHELIAL_CELL_SIGNALING_IN_HELICOBACTER_PYLORI_INFECTION	68	16	6	0.874
VASOPRESSIN-REGULATED_WATER_REABSORPTION	44	12	6	0.872
LONG-TERM_DEPRESSION	70	17	6	0.865
PHAGOSOME	154	21	6	0.864
SPLICEOSOME	127	15	6	0.853

© 2021. Kim H et al. Published online at http://www.aginganddisease.org/EN/10.14336/AD.2021.0621

RNA_TRANSPORT	144	27	6	0.845
PURINE_METABOLISM	161	15	6	0.816
CYSTEINE_AND_METHIONINE_METABOLISM	36	6	6	0.799
OLFACTORY_TRANSDUCTION	388	7	3	0.779
HUNTINGTON'S_DISEASE	183	24	6	0.76
ACUTE_MYELOID_LEUKEMIA	57	12	6	0.742
LEUKOCYTE_TRANSENDOTHELIAL_MIGRATION	116	19	6	0.733
SALIVARY_SECRETION	89	21	6	0.729
HEMATOPOIETIC_CELL_LINEAGE	88	15	6	0.721
PANCREATIC_SECRETION	103	19	6	0.715
RNA_DEGRADATION	57	12	5	0.683
STARCH_AND_SUCROSE_METABOLISM	53	8	5	0.683
THYROID_CANCER	29	10	5	0.664
LYSINE_DEGRADATION	44	9	6	0.626
PARKINSON'S_DISEASE	130	9	5	0.622
PENTOSE_PHOSPHATE_PATHWAY	26	2	1	0.62
HOMOLOGOUS_RECOMBINATION	28	2	1	0.594
AUTOIMMUNE_THYROID_DISEASE	52	5	4	0.56
CARDIAC_MUSCLE_CONTRACTION	77	14	6	0.557
GLYCOLYSIS_GLUCONEOGENESIS	65	5	4	0.554
SYSTEMIC_LUPUS_ERYTHEMATOSUS	136	11	4	0.554
VIRAL_MYOCARDITIS	70	9	6	0.522
O-GLYCAN_BIOSYNTHESIS	30	7	4	0.512
PPAR_SIGNALING_PATHWAY	70	10	5	0.499
ABC_TRANSPORTERS	44	7	6	0.49
COMPLEMENT_AND_COAGULATION_CASCADES	69	8	6	0.487
ALANINE_ASPARTATE_AND_GLUTAMATE_METABOLISM	32	5	5	0.481
BASAL_CELL_CARCINOMA	55	13	5	0.474
AMINO_SUGAR_AND_NUCLEOTIDE_SUGAR_METABOLISM	47	6	5	0.449
INTESTINAL_IMMUNE_NETWORK_FOR_IGA_PRODUCTION	48	8	4	0.443
PRIMARY_IMMUNODEFICIENCY	35	7	5	0.437
GLYCEROPHOSPHOLIPID_METABOLISM	79	14	4	0.431

CARBOHYDRATE_DIGESTION_AND_ABSORPTION	43	5	5	0.427
OXIDATIVE_PHOSPHORYLATION	132	7	5	0.425
BASAL_TRANSCRIPTION_FACTORS	35	5	4	0.417
RETINOL_METABOLISM	65	6	4	0.417
LYSOSOME	121	16	5	0.408
VALINE_LEUCINE_AND_ISOLEUCINE_DEGRADATION	44	5	5	0.407
PEROXISOME	79	7	3	0.401
N-GLYCAN_BIOSYNTHESIS	49	11	3	0.398
PATHOGENIC_ESCHERICHIA_COLI_INFECTION	56	7	4	0.384
STAPHYLOCOCCUS_AUREUS_INFECTION	55	4	4	0.381
VIBRIO_CHOLERAE_INFECTION	54	10	4	0.361
SNARE_INTERACTIONS_IN_VESICULAR_TRANSPORT	36	8	3	0.349
CYTOSOLIC_DNA-SENSING_PATHWAY	56	3	3	0.34
NUCLEOTIDE_EXCISION_REPAIR	44	3	3	0.334
SPHINGOLIPID_METABOLISM	40	9	4	0.331
TYROSINE_METABOLISM	41	3	2	0.329
BUTANOATE_METABOLISM	30	2	2	0.312
DNA_REPLICATION	36	3	2	0.307
HEDGEHOG_SIGNALING_PATHWAY	56	10	4	0.303
GLYCOSPHINGOLIPID_BIOSYNTHESIS_LACTO_AND_NEOLACTO_SERIES	26	6	4	0.302
STEROID_HORMONE_BIOSYNTHESIS	56	7	3	0.287
FRUCTOSE_AND_MANNOSE_METABOLISM	34	2	2	0.279
GLYCEROLIPID_METABOLISM	49	7	3	0.268
METABOLISM_OF_XENOBIOTICS_BY_CYTOCHROME_P450	71	6	3	0.265
PROPANOATE_METABOLISM	32	1	1	0.256
ANTIGEN_PROCESSING_AND_PRESENTATION	76	8	4	0.229
SELENOAMINO_ACID_METABOLISM	26	4	4	0.217
REGULATION_OF_AUTOPHAGY	34	5	4	0.211
PROTEASOME	44	6	4	0.21
COLLECTING_DUCT_ACID_SECRETION	27	3	3	0.169
ARGININE_AND_PROLINE_METABOLISM	54	4	3	0.164

© 2021. Kim H et al. Published online at http://www.aginganddisease.org/EN/10.14336/AD.2021.0621

TASTE_TRANSDUCTION	52	4	3	0.162
CITRATE_CYCLE_(TCA_CYCLE)	31	4	3	0.16
TRYPTOPHAN_METABOLISM	42	3	3	0.153
PHOTOTRANSDUCTION	29	2	3	0.147
ETHER_LIPID_METABOLISM	35	3	3	0.146
PENTOSE_AND_GLUCURONATE_INTERCONVERSIONS	31	4	2	0.144
ARACHIDONIC_ACID_METABOLISM	57	4	3	0.143
PYRIMIDINE_METABOLISM	99	2	2	0.137
DRUG_METABOLISM_OTHER_ENZYMES	52	4	2	0.136
ASCORBATE_AND_ALDARATE_METABOLISM	26	3	1	0.13
GLUTATHIONE_METABOLISM	50	5	3	0.126
PYRUVATE_METABOLISM	41	2	2	0.121
GLYCOSYLPHOSPHATIDYLINOSITOL(GPI)-ANCHOR_BIOSYNTHESIS	25	1	2	0.12
ASTHMA	30	3	3	0.117
MATURITY_ONSET_DIABETES_OF_THE_YOUNG	25	3	2	0.101
PORPHYRIN_AND_CHLOROPHYLL_METABOLISM	43	3	1	0.09
DRUG_METABOLISM_CYTOCHROME_P450	73	4	1	0.087
HISTIDINE_METABOLISM	29	2	1	0.071
GLYCINE_SERINE_AND_THREONINE_METABOLISM	32	1	1	0.066
GLYCOSAMINOGLYCAN_BIOSYNTHESIS_HEPARAN_SULFATE	26	2	1	0.056
AMINOACYL-TRNA_BIOSYNTHESIS	63	1	1	0.046
BASE_EXCISION_REPAIR	33	1	1	0.037
FATTY_ACID_METABOLISM	43	1	1	0.031

© 2021. Kim H et al. Published online at http://www.aginganddisease.org/EN/10.14336/AD.2021.0621
